# Polymorphisms at microRNA binding sites of Ara-C and anthracyclines-metabolic pathway genes are associated with outcome of acute myeloid leukemia patients

**DOI:** 10.1186/s12967-017-1339-9

**Published:** 2017-11-15

**Authors:** Hai-xia Cao, Chao-feng Miao, Liang Yan, Ping Tang, Li-rong Zhang, Ling Sun

**Affiliations:** 1 Department of Hematology, The First Affiliated Hospital of Zhengzhou University, No. 1 Jianshedong Road, Zhengzhou, 450052 Henan China; 2 Department of Vascular Surgery, The First Affiliated Hospital of Zhengzhou University, Zhengzhou, 450052 Henan China; 3 Department of Pharmacy, The First Affiliated Hospital of Zhengzhou University, Zhengzhou, 450052 Henan China; 4 Department of Hematology, The First Affiliated Hospital of Zhengzhou University, Zhengzhou, 450052 Henan China; 5 Department of Pharmacology, School of Basic Medical Sciences, Zhengzhou University, Zhengzhou, 450052 China

**Keywords:** Polymorphisms, MicroRNA-binding sites, Acute myeloid leukemia, Ara-C, Anthracyclines

## Abstract

**Background:**

Gene polymorphisms at microRNA-binding sites (poly-miRTS) may affect gene transcription and expression through miRNA regulation, which is associated with cancer susceptibility, sensitivity to chemotherapy and prognosis. This study investigated the association between poly-miRTS of Ara-C/anthracycline metabolic pathways genes and the outcome of acute myeloid leukemia (AML) in Chinese patients after Ara-C-based chemotherapy.

**Methods:**

A total of 17 poly-miRTS were selected from the SNPinfo Web Server and genotyped in 206 Chinese Han non-FAB-M3 AML patients using the SEQUENOM Mass-ARRAY system.

**Results:**

Among these 17 poly-miRTS, five Ara-C metabolic gene single nucleotide polymorphisms (SNPs, NT5C2 rs10786736 and rs8139, SLC29A1 rs3734703, DCTD rs7278, and RRM1 rs1042919) were identified to significantly associate with complete AML remission and/or overall and relapse-free survival (OS and RFS, respectively), and four anthracycline-metabolic gene SNPs (ABCC1 rs3743527, rs212091, and rs212090 and CBR1 rs9024) were significantly associated with chemotherapy-related toxicities. Moreover, SLC29A1 rs3734703 was shown to associate with both chemotherapy response and survival (adjusted OR 2.561 in the overdominant model; adjusted HR 2.876 for OS and 2.357 for RFS in the dominant model).

**Conclusions:**

The data from the current study demonstrated that the poly-miRTS of Ara-C/anthracyclines metabolic genes predicted the sensitivity and side effects of AML to Ara-C-based chemotherapy and patient survival. Further study will confirm them as biomarkers for AML patients after Ara-C-based chemotherapy.

**Electronic supplementary material:**

The online version of this article (10.1186/s12967-017-1339-9) contains supplementary material, which is available to authorized users.

## Background

Acute myeloid leukemia (AML) is a rapidly growing hematologic malignancy and also the most commonly occurring acute leukemia in adults [1]. AML heterogeneity affects treatment and prognosis, and a combination of cytarabine (Ara-C) with different anthracyclines has been the most common treatment for adult AML patients. However, there are significant inter-patient differences in treatment response and toxicity, and only 20–30% of AML patients can reach long-term disease-free survival, whereas the majority of patients die due to persistent or relapsed AML [1]. To date, resistance to Ara-C or anthracycline is one of the most important reasons for chemotherapeutic failure in AML patients [2, 3] for reasons that remain to be defined. For example, hereditary factors represent one of the determinants for chemotherapy efficacy and side effects. Genetic variants, particularly single-nucleotide polymorphisms (SNPs) of Ara-C and anthracycline metabolic genes, have been identified as determinants for treatment responses and side effects [4–6]. These previous studies primarily focused on common gene SNPs in the coding regions; however, these may not consider the role of the gene 3′-untranslational region (3′-UTR), which may affect post-transcriptional gene regulation. For example, microRNAs (miRNAs) are a class of small endogenous non-coding RNA that regulate gene expression through binding to the 3′-UTR of their target mRNAs [7, 8]. A growing number of studies have suggested that gene polymorphisms at microRNA binding sites (poly-miRTS) are closely related to cancer susceptibility, sensitivity to chemotherapy, and patient prognosis [9, 10], indicating that these SNPs could be a useful novel class of genetic variations for further investigation and that they may potentially become novel markers in predicting treatment responses and patient survival. Mishra et al. [11] in 2007 first identified a poly-miRTS that was associated with pharmacogenomics. Thus, further study of the poly-miRTS in genes that are related to drug metabolism could help medical oncologists better select treatment regimens and predict treatment responses.

Based on this postulation, we therefore hypothesized that poly-miRTS in Ara-C and anthracycline-metabolic pathway genes could predict response to and prognosis of Ara-C-based chemotherapy in Chinese AML patients. In this study, we searched the public databases and identified SNPs at the 3′-UTR of those genes, which may affect miRNA binding and consequently influence their expression and genotyped 17 of these SNPs in 206 blood samples from Chinese AML patients. We then evaluated and associated them with survival, chemotherapy response and toxicity in AML patients after Ara-C-based chemotherapy.

## Methods

### Study population

A total of 206 patients with de novo AML (diagnosed according to the WHO criteria) other than M3 were treated in The First Affiliated Hospital of Zhengzhou University (Zhengzhou, China) between January 2012 and December 2016. The patients that were diagnosed with any other cancers or previously administered cytotoxic drugs or radiation were excluded from this study. Clinical data from these patients were collected from their medical records, and patients were followed up until April 2, 2017 via telephone review. All participants were Han Chinese. This study was approved by the Ethical Committees of The First Affiliated Hospital of Zhengzhou University (Zhengzhou, China), and informed consent was obtained from each patient or their family members.

### Chemotherapy regimens

All patients received Ara-C-based standard chemotherapy regimens for induction therapy. Among them, 89 patients for DA (45 mg/m^2^ daunorubicin for 1–3 days and 100 mg/m^2^ Ara-C for 1–7 days), 61 for IA (10 mg/m^2^ idarubicin for 1–3 days and 100 mg/m^2^ Ara-C for 1–7 days), 10 for MA (4 mg/m^2^ mitoxantrone for 1–5 days and 100 mg/m^2^ Ara-C for 1–7 days), 27 for TA (pirarubicin 40 mg/day for 1–3 days and 100 mg/m^2^ Ara-C for 1–7 days), and 19 for CAG (200 µg/m^2^ G-CSF for 1–14 days, 5–7 mg/m^2^ aclarubicin for 1–8 days, and 10 mg/m^2^Ara-C for 1–14 days). The patients received another cycle of induction chemotherapy if they did not achieve CR after the first regimen.

### Evaluation of chemotherapy response and toxicity

The treatment responses were evaluated after the second cycle of the induction chemotherapy regimen as complete remission (CR) or non-CR according to the International Working Group AML criteria [12]. The CR was defined as the following: blast cell counts in the bone marrow < 5%, absence of extramedullary disease; absolute neutrophil count > 1 × 10^9^/L, platelet count > 100 × 10^9^/L, and independence of red cell transfusion. Patients with other treatment responses, including partial remission, non-remission and early death were assigned to the non-CR group. Disease relapse was defined as the presence of more than 5% of blast cells in the bone marrow or the reappearance of blast cells in the blood or the development of extramedullary disease. Relapse-free survival (RFS) was evaluated by measurement from the date of remission until the date of relapse or death from any cause; patients not known to have relapsed or died at the last follow-up were censored on the date they were last followed up. The overall survival (OS) was measured from the date of diagnosis until death from any cause, with observation censored on the date the patient was last known to be alive or at the time of hematopoietic stem cell transplantation after their CR. The adverse events during chemotherapy were recorded and graded according to the National Cancer Institute Common Terminology Criteria for Adverse Events version 4.0 [13]. For data analysis, more than 2 grade adverse events were collected and analyzed.

### Identification and selection of poly-miRTS

In this study, we selected a total of 20 key candidate genes described in pervious studies [14–16] are directly related to the pharmacokinetic and/or pharmacodynamics pathways of Ara-C and anthracycline, i.e., 10 Ara-C associated genes (Deoxycytidine kinase*, DCK*; Cytidine deaminase*, CDA*; human equilibrative nucleoside transporter, *hENT1*/*SLC29A1*; Solute carrier family 28 member, *SLC28A3, SLC28A1*; 5′ nucleotidase 3*, NT5C3*; 5′ nucleotidase 2, *NT5C2*; Deoxycytidylate deaminase*, DCTD*; Ribonucleotide reductase*, RRM1* and *RRM2*) and 10 anthracycline-associated genes (ATP binding cassette subfamily A, B, C, G member, *ABCA3*, *ABCB1*, *ABCC1*, *ABCG2, ABCC10, ABCC11*; Glutathione S-transferase, *GSTM1, GSTT1*, Carbonyl reductases, C*BR3,* and *CBR1*). We then utilized tools in the SNPinfo Web Server to identify putative SNPs in 3′-UTR with potential miRNA-binding sites. A linkage disequilibrium value (R^2^ < 0.8) and minor allele frequency (MAF) ≥ 0.05 in the CHB (Chinese Han in Beijing) were further applied to filter these SNPs. We obtained a total of 17 SNPs in nine genes to be analyzed in this study (Table 1).

**Table 1:** Characteristics of 17Poly-miRTS in Ara-C/anthracyclines pathway genes

Gene	SNP ID	Chromosome location	Position^a^	Variants	MAF in CHB	Putative miRNA and effect^b^
*SLC29A1*	rs3734703	6	44233997	C>A	0.20	hsa-miR-1207-5p (break) hsa-miR-571 (decrease)
*NT5C2*	rs10786736	10	103089359	G>C	0.22	hsa-miR-581 (break) hsa-miR-1238 (enhance) hsa-miR-1236 (decrease)
	rs8139	10	103088366	T>C	0.50	hsa-miR-373 (break) hsa-miR-578 (break) hsa-miR-125a-3p (create)
	rs12573199	10	103089087	A>T	0.08	hsa-miR-325 (create) hsa-miR-548a-5p (break)
*DCTD*	rs3811810	4	182890990	G>A	0.08	hsa-miR-574-5p (create) hsa-miR-599 (create)
	rs7278	4	182890334	C>T	0.17	hsa-miR-935 (decrease)
	*rs851*	4	182890517	G>A	0.30	hsa-miR-1288 (create) hsa-miR-423-5p (enhance) hsa-miR-556-5p (create)
	rs9542	4	182891198	G>A	0.47	hsa-miR-1305 (break)
*RRM1*	rs1042919	11	4138534	T>A	0.32	hsa-miR-944 (enhance)
*SLC28A1*	rs8025045	15	84945341	G>T	0.06	hsa-miR-500 (create) hsa-miR-574-3p (decrease) hsa-miR-767-3p (decrease)
*CBR1*	rs9024	21	36073015	G>A	0.20	hsa-miR-944 (break) hsa-miR-325 (enhance)
*ABCB1*	rs3842	7	87504050	A>G	0.28	hsa-miR-548g (break)
*ABCC1*	rs212090	16	16142147	T>A	0.14	hsa-miR-1292 (break) hsa-miR-548j (break) hsa-miR-450b-3p (create) hsa-miR-769-3p (create) hsa-miR-199a-3p (decrease) hsa-miR-199b-3p (decrease)
	rs212091	16	16142793	A>G	0.26	hsa-miR-1303 (create)
	rs3743527	16	16141824	C>T	0.45	hsa-miR-625 (break) hsa-miR-141 (break) hsa-miR-760 (break) hsa-miR-1291 (enhance)
	rs4148380	16	16142574	G>A	0.05	hsa-miR-187 (break) hsa-miR-599 (create)
*NQO1*	rs10517	16	69709857	C>T	0.37	hsa-miR-1243 (create) hsa-miR-587 (create) hsa-miR-324-5p (enhance)

^a^Obtained from NCBI: Assembly: GRCh38.p7

^b^Obtained from https://snpinfo.niehs.nih.gov/

### Genotyping

Blood samples were collected into Ethylene Diamine Tetraacetic Acid tubes from patients prior to chemotherapy, and genomic DNA was extracted using the TIANamp Blood DNA Kit (TIANGEN, Beijing, China) and stored at − 80 °C until use. These 17 poly-miRTS were genotyped using the SEQUENOM Mass-ARRAY system with specific amplification primers and extension primers (Additional file 1: Table S1).

### Statistical analysis

Genotype deviations of the Hardy–Weinberg equilibrium were assessed using the Pearson Chi square test, while continuous data were converted into categorical data using their median. The significant difference in genotypes and clinical information between chemotherapy toxicity, CR and non-CR was calculated using Pearson/Continuity Correction Chi Square test or Fisher’s exact test. Odds ratios (ORs) and their 95% CIs were calculated to estimated the relative risk of responding to treatment using the logistic regression analysis while adjusting for age, risk stratifications and platelets. Kaplan–Meier curves and the log-rank test were performed to assess OS and RFS stratified by the genotypes of each SNP. Associations between the clinicopathological data and survival were also estimated. The hazard ratios (HR) and 95% CIs for OS and RFS were estimated using the Cox proportional hazards model while adjusting for risk stratifications. All statistical analyses were performed using SPSS 21.0 software (SPSS Inc., Chicago, IL, USA), and *P* < 0.05 in a two-sided test was considered to be statistically significant.

## Results

### Patient characteristics and treatment outcomes

The baseline characteristics and treatment results of all 206 AML patients are summarized in Table 2. Particularly, the median age of the patients was 43 years, ranging between 13 and 76 years, and the ratio of male to female patients was 102–104. The most frequent French–American–British (FAB) subtype of AML disease was M2 (50.5%) followed by M5 (22.3%). Overall, 176 patients (85.4%) achieved CR after Ara-C based induction chemotherapy. 125 (60.7%) patients achieved CR after their first course of induction therapy, and 51 (24.8%) patients achieved CR after two courses of induction therapy. Among these 206 patients, 21 (10.2%) relapsed during the follow-up period. The mean and median follow-up periods for these 206 patients were 475 and 288 days, respectively (range 22 and 1890 days), while 31 (15.0%) of 206 patients died at the end of the follow-up period.

**Table 2:** Patient characteristics and treatment outcomes

Characteristics	Patients, n (%)	Median (range)
Sex		
Male	101 (49.0)	
Female	105 (51.0)	
Age at diagnosis, year		43 (13–76)
≥ 43	106 (51.5)	
< 43	100 (48.5)	
BM blasts, %		67.2 (22–97.8)
≥ 67.2	100 (48.5)	
< 67.2	99 (48.1)	
Unknown	7 (3.4)	
WBC, ×109/L		20.7 (0.29–412.5)
≥ 20.7	104 (50.5)	
< 20.7	102 (49.5)	
Hemoglobin, g/L		81 (20–138)
≥ 81	103 (50.0)	
< 81	101 (49.0)	
Unknown	2 (1.0)	
Platelets, ×109/L	206	38.5 (5–542)
< 38.5	103 (50)	
≥ 38.5	103 (50)	
FAB classification		
M0	2 (1.0)	
M1	14 (6.8)	
M2	104 (50.5)	
M4	31 (15.0)	
M5	46 (22.3)	
M6	7 (3.4)	
M7	2 (1.0)	
Risk stratifications^a^		
Low risk	35 (17.0)	
Intermediate risk	118 (57.3)	
High risk	53 (25.7)	
*FLT3*-*ITD* mutation		
Positive/negative	25/181 (12.1/87.9)	
*NPM1* mutation		
Positive/negative	32/174 (15.5/84.5)	
*KIT* mutation		
Positive/negative	9/197 (4.4/95.6)	
*CEBPA* mutation		
Positive/negative	7/199 (3.4/96.6)	
Karyotype		
Normal	133 (64.6)	
inv (16)	8 (3.9)	
t (8;21)	11 (5.3)	
t (6;9)	3 (1.5)	
t (9;22)	1 (0.5)	
t (9;11)	2 (1.0)	
inv (3)	1 (0.5)	
+ 8	2 (1.0)	
− 7	1 (0.5)	
Other	44 (21.4)	
Treatment response		
CR	176 (85.4)	
CR after 1st course	125 (60.7)	
CR after 2nd course	51 (24.8)	
Non-CR	30 (14.6)	
Treatment outcome		
Death	31 (15.0)	
Relapse	21 (10.2)	
Adverse events (grade ≥ 2)		
Myelosuppression	168 (81.6)	
Liver function damage	23 (11.2)	
Cardiotoxicity	11 (5.3)	
Gastrointestinal reaction	66 (32.0)	

*BM* bone marrow, *WBC* white blood cell, *FAB* French–American–British

^a^Classified according to NCCN guidelines version 1.2015 acute myeloid leukemia

### Association between clinical characteristics and chemotherapy response and toxicity, OS, and RFS

Clinical characteristics, such as platelets counts and risk stratification, have been significantly associated with chemotherapy response and toxicity, OS, and RFS (Table 3). Platelet counts at diagnosis were a unique clinical characteristic that was significantly associated with response to chemotherapy (*P* = 0.006); the patients with higher platelet counts had a higher CR ratio (92.2% vs. 78.6%, OR 3.225, 95% CI 1.362–7.635). The patients with platelet counts > 38.5 × 10^9^/L had a longer mean OS than the patients with counts < 38.5 × 10^9^/L (1525 days vs. 1290 days); however, this did not reach statistical significance (*P* = 0.057).

**Table 3:** Significant effect of clinical variables on clinical outcomes

Clinical variables	Clinical outcomes	*P*	OR/HR (95% CI)
Risk stratifications	OS (mean day ± SE)	0.027	
Low + intermediate	1473 ± 82		1.00 (reference)
High	1048 ± 113		2.325 (1.103–4.900)
Risk stratifications	PFS (mean day ± SE)	0.013	
Low	1532 ± 151		1.00 (reference)
Intermediate	1096 ± 99	0.056	2.762 (0.974–7.834)
High	740 ± 105	0.005	4.801 (1.606–14.350)
Platelets, ×109/L	CR (%)		
< 38.5	78.6		1.00 (reference)
≥ 38.5	92.2	0.006	3.225 (1.362–7.635)
Risk stratifications	Liver function damage (%)	0.009	
Low	22.9		1.00 (reference)
Intermediate	5.9	0.008	0.213 (0.071–0.638)
High	15.1	0.355	0.660 (0.202–1.784)
Risk stratifications	Cardiotoxicity (%)	0.004	
Low	17.1		1.00 (reference)
Intermediate	2.5	0.005	0.126 (0.030–0.535)
High	3.8	0.079	0.190 (0.036–1.001)

*CR* complete remission, *OS* overall survival, *RFS* relapse-free survival, *CI* confidence interval, *HR* hazard ratio, *OR* odd risk, *SE* standard error

Risk stratification was the only clinical factor associated with chemotherapy toxicity in Ara-C-based treatment. Compared with the low-risk patients, the intermediate risk patients had a lower risk of liver function damage and cardiotoxicity (*P* = 0.008, OR 0.213, 95% CI 0.071–0.638; *P* = 0.005, OR 0.126, 95% CI 0.030–0.535, respectively). Furthermore, the risk stratification was also associated with the OS (*P* = 0.022) and RFS (*P* = 0.008). Because there was no mortality in the low risk group, we put the low and intermediate risk patients together to carry out the OS analysis. Our data showed that compared with the low and intermediate risk groups, the high risk patients had a relatively higher risk of death [the mean OS, 1048 days vs. 1473 days, *P* = 0.027, HR 2.325, 95% CI (1.103–4.900)]; the high risk patients compared with low risk also had poorer RFS (mean RFS of 740 days vs. 1532 days, HR 4.821, 95% CI (1.611–14.427).

However, other clinical characteristics, such as gender, age, WBC, hemoglobin count, and BM blast percent at diagnosis showed no significant differences in CR ratio, chemotherapy toxicity or survival rates (Additional file 2: Table S2, Additional file 3: Table S3).

### Associations between poly-miRTS and response to chemotherapy

The allele frequencies and genotype distributions of these 17 poly-miRTS are summarized in Additional file 4: Table S4 with the Hardy–Weinberg equilibrium. Patient characteristics, such as chemotherapy regimens or risk groups, showed no significant differences according to the genotypes of these SNPs (Additional file 5: Table S5). Associations between poly-miRTS and chemotherapy response are shown in Additional file 4: Table S4. Specifically, 4 of 17 poly-miRTS (*SLC29A1* rs3734703, *DCTD* rs3811810 and rs7278, and *RRM1* rs1042919) were significantly associated with CR (Table 4). The Rs3734703 CC+AA genotypes had a higher CR ratio than the CA genotype (89.2% vs. 77.6%, *P* = 0.027 in the overdominant model). A higher CR ratio also occurred in the rs3811810 AA+GA genotypes (97.4% vs. 82.6%, *P* = 0.018 in the dominant model) and in the rs7278 TT+TC genotypes (97.6% vs. 82.4%, *P* = 0.014 in the dominant model), while Rs1042919 AA+AT showed a lower CR ratio compared with the TT genotype (77.3% vs. 92.7%, *P* = 0.002 in the dominant model).

**Table 4:** Significant effects of poly-miRTS on chemotherapy response in AML patients by Univariate and Logistic regression analysis

Genotype	Total (N)	CR, N (%)	*P*	OR (95% CI)	*P* ^a^	OR (95% CI)^a^
rs3811810						
GG	167	138 (82.6)		1.00 (reference)		1.00 (reference)
AA	3	3 (100)	0.999	–		–
GA	36	35 (97.2)	0.054	7.355 (0.968–55.873)	0.070	6.671 (0.854–52.098)
AA+GA^b^	39	38 (97.4)	0.044	7.986 (1.053–60.532)	0.058	7.258 (0.934–56.388)
rs7278						
CC	165	136 (82.4)		1.00 (reference)		1.00 (reference)
TT	4	4 (100)	0.999	–		–
TC	37	36 (97.3)	0.049	7.676 (1.011–58.276)	0.053	7.588 (0.971–59.319)
TT+TC^b^	41	40 (97.6)	0.038	8.529 (1.127–64.581)	*0.040*	8.572 (1.106–66.423)
rs1042919						
TT	109	101 (92.7)		1.00 (reference)		1.00 (reference)
AA	9	7 (77.8)	0.146	0.277 (0.049–1.561)	0.060	0.165 (0.025–1.078)
AT	88	68 (77.3)	0.003	0.269 (0.112–0.646)	*0.003*	0.253 (0.102–0.630)
AA+AT^b^	97	75 (77.3)	0.003	0.270 (0.114–0.640)	*0.002*	0.246 (0.100–0.604)
rs3734703						
CC	122	108 (88.5)		1.00 (reference)		1.00 (reference)
AA	17	16 (94.1)	0.495	2.074 (0.255–16.863)	0.495	2.097 (0.249–17.630)
CA	67	52 (77.6)	0.050	0.449 (0.202–1.000)	0.066	0.455 (0.197–1.054)
CC+AA/CA^c^	139	124 (89.2)	0.030	2.385 (1.087–5.231)	*0.023*	2.561 (1.136–5.775)

Italic values indicate statistical significance

^a^Adjusted for age, risk stratifications and platelets

^b^The dominant model

^c^The overdominance model

We then performed a logistic regression analysis for associations between the above four poly-miRTS and CR ratio after adjusting for age, risk stratifications, and platelets count. We found that only three poly-miRTS (rs3734703, rs7278, and rs1042919) were significantly associated with chemotherapy response (Table 4). In particular, a higher CR ratio occurred in patients with the rs3734703 CC+AA genotypes (*P* = 0.023 in the overdominant model) with an OR of 2.561 (95% CI 1.136–5.775) or rs7278 TT+TC genotypes (*P* = 0.040 in the dominant model) with an OR of 8.572 (95% CI 1.106–66.423). However, patients with rs1042919 AA+AT showed a lower CR ratio (*P* = 0.002 in the dominant model) with an OR of 0.246 (95% CI 0.100–0.604).

### Association between poly-miRTS and chemotherapy toxicity

There were only four poly-miRTS in anthracyclines pathways associated with chemotherapy toxicity (Table 5). In brief, *ABCC1* rs3743527 TT vs. CC had a higher risk of developing myelosuppression (*P* = 0.007, OR 10.625, 95% CI 1.339–84.317), whereas *ABCC1* rs212091 GG+AG vs. AA had a lower risk of developing myelosuppression (*P* = 0.003, OR 0.350, 95% CI 0.171–0.719). *ABCC1* rs212090 AT vs. TT was associated with a higher risk of gastrointestinal reaction (*P* = 0.010, OR 2.259, 95% CI 1.211–4.213), while *CBR* rs9024 AG+AA vs. GG had a higher risk of cardiotoxicity (*P* = 0.010, OR 7.358, 95% CI 1.547–34.989). However, there were no significant differences in the cardiotoxicity, gastrointestinal reactions, liver function damage or myelosuppression among genotypes of the remaining 13 polymorphisms (all *P* > 0.05; Additional file 6: Table S6).

**Table 5:** Significant effects of poly-miRTS on adverse events after chemotherapy of AML patients (Chi square/Fisher’s exact test)

SNP genotypes	Total (n)	Adverse events			OR (95% CI)
		Yes (n, %)	No (n)		
rs3743527		Myelosuppression			
CC	63	48 (76.2)	15		1.00 (reference)
TC	108	85 (78.7)	23	0.703	1.155 (0.51–2.422)
TT	35	34 (97.1)	1	*0.007*	10.625 (1.339–84.317)
TC+TT^a^	143	119 (83.2)	24	0.236	1.549 (0.749–3.206)
rs212091		Myelosuppression			
AA	122	107 (87.7)	15		1.00 (reference)
AG	72	51 (70.8)	21	*0.003*	0.340 (0.162–0.715)
GG	12	9 (75.0)	3	0.431	0.421 (0.102–1.729)
AG+GG^a^	84	60 (71.4)	24	*0.003*	0.350 (0.171–0.719)
rs212090		Gastrointestinal reaction			
TT	135	37 (27.4)	98		1.00 (reference)
AT	63	29 (46.0)	34	*0.010*	2.259 (1.211–4.213)
AA	8	0 (0)	8	0.192	–
AA+AT^a^	71	29 (40.8)	42	0.049	1.829 (0.998–3.35)
rs9024		Cardiotoxicity			
GG	123	2 (1.6)	121		1.00 (reference)
GA	69	7 (10.1)	62	*0.020*	6.831 (1.378–33.866)
AA	14	2 (14.3)	12	0.052	10.083 (1.301–78.151)
GA+AA^a^	83	9 (10.8)	74	*0.010*	7.358 (1.547–34.989)

Italic values indicate statistical significance

^a^The dominant model

### Association between poly-miRTS and survival of AML patients

**Fig. 1:**
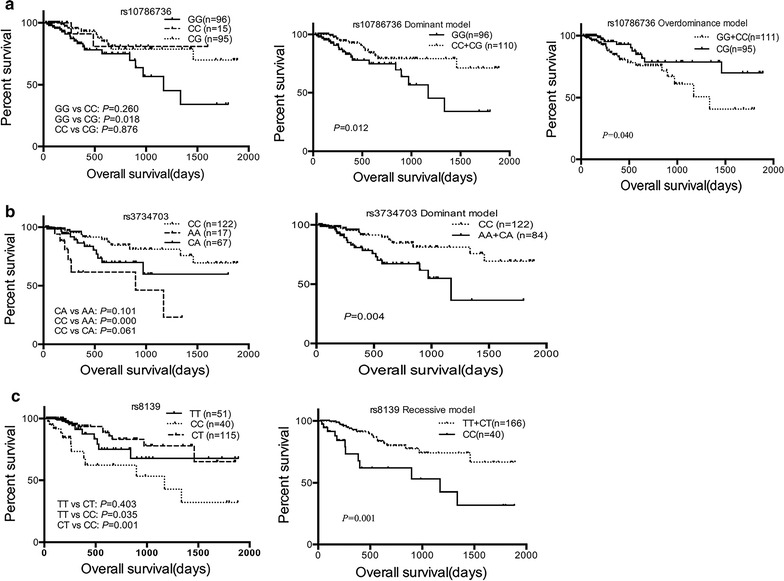
Significant effects of poly-miRTS on OS of AML patients by the Kaplan–Meier method and log-rank test. **a** rs10786736; **b** rs3734703; **c** rs8139

**Fig. 2:**
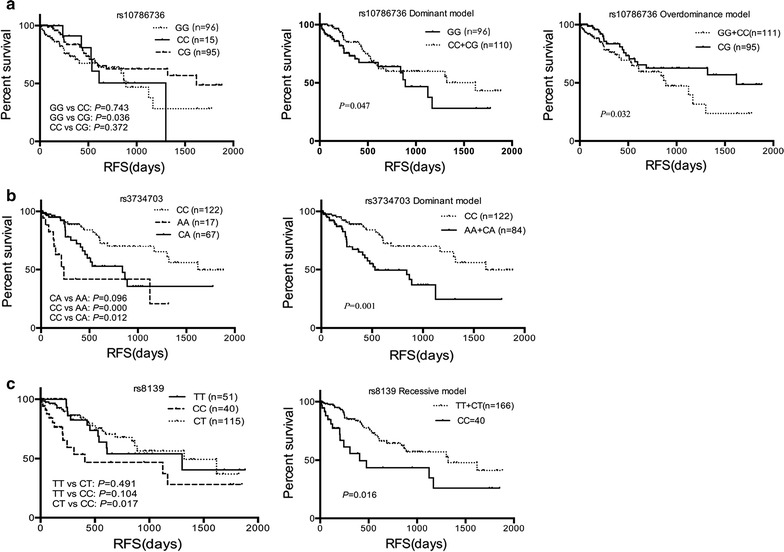
Significant effect of poly-miRTS on RFS of AML patients by the Kaplan–Meier method and log-rank test. **a** rs10786736; **b** rs3734703; **c** rs8139

The Kaplan–Meier curves with the log rank test showed that there was a significant association between *SLC29A1* rs3734703 and *NT5C2* rs10786736 and rs8139poly-miRTS with survival (Figs. 1, 2). The multivariable analysis using Cox regression after adjusting for risk stratifications showed that rs3734703, rs10786736, and rs8139 poly-miRTS were independent predictors for OS (Table 6) and RFS (Table 7). Both rs3734703 AA+CA genotypes and rs10786736 GG+CC genotypes were associated with shorter OS (analyzed using the dominant model with an adjusted *P* = 0.006, adjusted HR 2.876, and 95% CI 1.364–6.062; and in the overdominant model with the adjusted *P* = 0.009, HR 2.837, and 95% CI 1.294–6.219) and shorter RFS analyzed using the dominant model with the adjusted *P* = 0.003, adjusted HR 2.357, and 95% CI 1.337–4.155 and the overdominant model with the adjusted *P* = 0.022, HR 1.957, and 95% CI 1.101–3.478. In addition, the rs10786736 CG+CC genotypes associated with longer OS were analyzed using the dominant model (with an adjusted *P* = 0.012, adjusted HR 0.392, and 95% CI 0.189–0.815) and RFS using the dominant model (with an adjusted *P* = 0.012, adjusted HR 0.392, and 95% CI 0.189–0.815) in comparison with the GG genotype, whereas the rs8139 CC genotypes compared with the TT+CT genotypes were associated with shorter OS and RFS using the recessive model (with an adjusted *P* = 0.001, adjusted HR 3.475, 95% CI 1.674–7.213 and adjusted *P* = 0.015, HR 2.172, 95% CI 1.162–4.059, respectively). However, the other 13 poly-miRTS had no significant associations with OS or RFS (all *P* > 0.05) (Additional file 7).

**Table 6:** Significant effects of poly-miRTS on OS of AML patients by univariate and multivariate Cox regression analysis

Genotype	Mean ± SE (day)	P	RR (95% CI)	P^a^	RR (95% CI)^a^
rs10786736					
GG	1134 ± 115		1.00 (reference)		1.00 (reference)
CC	1367 ± 149	0.236	0.414 (0.096–1.780)	0.253	0.426 (0.099–1.836)
CG	1550 ± 95	0.022	0.404 (0.186–0.876)	*0.016*	0.386 (0.177–0.839)
CC+CG^b^	1560 ± 85	0.015	0.406 (0.196–0.841)	*0.012*	0.392 (0.189–0.815)
GG+CC^c^	1192 ± 107	0.045	2.178 (1.018–4.660)	*0.033*	2.297 (1.070–4.933)
rs3734703					
CC	1571 ± 83		1.00 (reference)		1.00 (reference)
AA	801 ± 146	0.001	5.121 (1.985–13.214)	*0.000*	5.877 (2.244–15.39)
CA	1290 ± 127	0.056	2.231 (0.981–5.073)	0.057	2.219 (0.975–5.050)
AA+CA^b^	1100 ± 133	0.006	2.842 (1.350–5.980)	*0.005*	2.902 (1.377–6.117)
rs8139					
TT	1440 ± 137		1.00 (reference)		1.00 (reference)
CC	1039 ± 166	0.042	2.538 (1.033–6.236)	0.060	2.370 (0.963–5.831)
CT	1526 ± 93	0.391	0.671 (0.270–1.669)	0.204	0.547 (0.215–1.388)
CC/CT		0.002	3.782 (1.662–8.607)	*0.001*	4.334 (1.885–9.964)
TT+CT^d^	1510 ± 78	0.001	3.259 (1.574–6.746)	*0.001*	3.475 (1.674–7.213)

Italic values indicate statistical significance

^a^Adjusted for risk stratifications, high risk vs. low + intermediate risk

^b^The dominant model

^**c**^The overdominance model

^d^The recessive model

**Table 7:** Significant effects of poly-miRTS on RFS of AML patients by univariate and multivariate Cox regression analysis

Genotype	Mean ± SE (day)	*P*	HR (95% CI)	*P* ^a^	HR (95% CI)^a^
rs10786736					
GG	925 ± 113		1.00 (reference)		1.00 (reference)
CC	892 ± 146	0.722	0.851 (0.350–2.070)	0.926	0.959 (0.393–2.342)
CG	1267 ± 10	0.032	0.523 (0.289–0.946)	*0.026*	0.507 (0.279–0.921)
CC+CG^b^	1214 ± 99	0.050	0.577 (0.333–1.000)	*0.049*	0.574 (0.331–0.997)
CC+GG^c^	912 ± 97	0.034	1.855 (1.047–3.287)	*0.022*	1.957 (1.101–3.478)
rs3734703					
CC	1325 ± 96		1.00 (reference)		1.00 (reference)
AA	592 ± 157	0.000	4.257 (1.936–9.360)	*0.000*	4.615 (2.086–10.209)
CA	921 ± 123	0.014	2.158 (1.169–3.984)	*0.032*	1.955 (1.058–3.611)
AA+CA^b^	815 ± 113	0.001	2.544 (1.446–4.478)	*0.003*	2.357 (1.337–4.155)
rs8139					
TT	1088 ± 150		1.00 (reference)		1.00 (reference)
CC	834 ± 172	0.122	1.801 (0.855–3.796)	0.175	1.677 (0.794–3.541)
CT	1182 ± 100	0.482	0.789 (0.408–1.526)	0.249	0.673 (0.343–1.319)
CC/CT		0.015	2.282 (1.174–4.435)	*0.007*	2.537 (1.295–4.970)
TT+CT^d^	1176 ± 86	0.018	2.105 (1.134–3.907)	*0.015*	2.172 (1.162–4.059)

Italic values indicate statistical significance

^a^Adjusted for risk stratifications

^b^The dominant model

^**c**^The overdominance model

^d^The recessive model

### Combined effects of SNPs on chemotherapy response, OS, and RFS

We found that rs10786736, rs8139, rs3734703, rs7278, and rs1042919 had significant associations with Chinese AML chemotherapy response and/or OS and RFS individually. To analyze the combined effects of multi-locus SNPs on AML prognosis, we created a combined genotype score model [17] using the defined genotype score, i.e., favorable genotypes (rs7278 TT/TC genotype, rs3734703 CC/AA genotype, or rs1042919 TT genotype for CR; rs10786736 CC/CG genotype, rs3734703 CC genotype, or rs8139 TT/CT genotype for OS and RFS) defined as score 1 and unfavorable genotypes (i.e., rs7278 CC genotype, rs734693 CA genotype, or rs1042919 AA/AT genotype for CR; rs10786736 GG genotype, rs3734703 AA/CA genotype, or rs8139 CC genotype for OS and RFS) defined as score 0.

A defined favorable response group (composite score 1, 2, or 3) in which patients have at least one favorable genotype had a higher CR ratio compared with a defined unfavorable response group (composite score 0) in which patients have three unfavorable genotypes (85.4% vs. 68.8%, *P* = 0.005). After adjusting for age, risk stratifications and platelets, binary logistic regression analysis indicated that the defined favorable response group still had a higher CR ratio (OR = 3.624; 95% CI 1.398–9.395; P = 0.008) (Table 8).

**Table 8:** Combined effects of rs7278, rs3734703 and rs1042919 genotypes on chemotherapy response in AML patients

Composite score^a^	N	CR (%)	*P*	*P* ^b^	OR (95% CI)^b^
0	32	68.8			1.00 (reference)
1	77	79.2	0.246	0.270	1.767 (0.642–4.865)
2	79	94.9	0.001	0.001	8.604 (2.318–31.93)
3	18	100	0.998	0.998	–
1 + 2 + 3	174	85.4	0.005	0.008	3.624 (1.398–9.395)

^a^Combined genotype score model was created by compiling the genotyped data of SNPs rs7278, rs3734703 and rs1042919. Score 1 indicated favorable genotypes (i.e., rs7278 TT/TC genotype, rs3734703 CC/AA genotype, or rs1042919 TT genotype) and a score of 0 indicated unfavorable genotypes (i.e., rs7278 CC genotype, rs734693 CA genotype, or rs1042919 AA/AT genotype). After adding up these scores, four composite score groups were generated: composite score 0, 1, 2 and 3

^b^Adjusted for age, risk stratifications and platelets

**Fig. 3:**
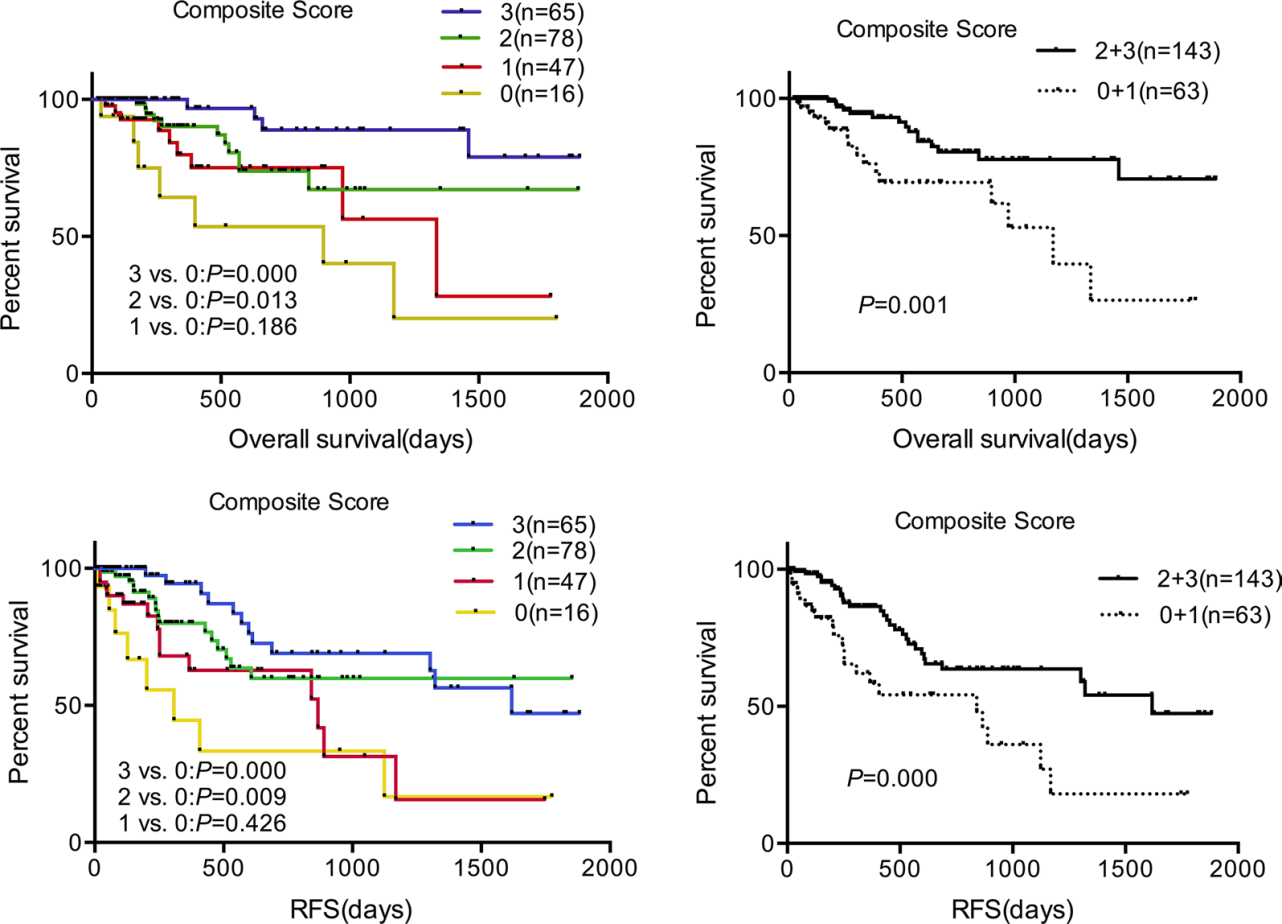
Combined effects of rs10786736, rs3734703 and rs8139 genotypes on survival of AML patients analyzed by the Kaplan–Meier method and log-rank test. A combined genotype score model was created by compiling the genotyped data of SNPs rs10786736, rs3734703 and rs8139. Score 1 indicated favorable genotypes (i.e., rs10786736 CC/CG genotype, rs3734703 CC genotype, or rs8139 TT/CT genotype), and score 0 indicated unfavorable genotypes (i.e., rs10786736 GG genotype, rs3734703 AA/CA genotype, or rs8139 CC genotype). After adding up these scores, four composite score groups were generated: composite score 0, 1, 2 and 3

A defined favorable prognosis group (including a composite score of 2 or 3) in which patients have at least two favorable genotypes showed longer OS and RFS than a defined unfavorable prognosis group (including a composite score of 0 or 1) in which patients have at least two unfavorable genotypes (1037 days vs. 1551 days, P = 0.001 for OS; 762 days vs. 1264 days, P = 0.000 for RFS, Fig. 3). After adjusting for risk stratifications, multivariate analysis indicated that the favorable prognosis group appeared to be an independent predictive factor for longer OS and RFS (HR = 0.316, 95% CI 0.155–0.642, P = 0.001 for OS; HR = 0.389, 95% CI 0.224–0.675, P = 0.001 for RFS, Additional file 8: Table S7).

## Discussion

To the best of our knowledge, the current study is the first to evaluate and associate the poly-miRTS of pharmacogenomics-related genes in the metabolism of Ara-C and anthracyclines with AML treatment responses and outcome. We identified five poly-miRTS in Ara-C-metabolic genes (*NT5C2* rs10786736 and rs8139, *SLC29A1* rs3734703, *DCTD* rs7278, and *RRM1* rs1042919) to be significantly associated with complete remission after AraC-based chemotherapy and/or OS and RFS in these Chinese AML patients. The combined effects of these SNPs showed that patients with more unfavorable genotypes had worse prognosis. The combination of multi-locus SNPs could improve the detection power of genetic effects associated with the treatment outcomes. We also found that four poly-miRTS in anthracyclines-metabolic genes (*ABCC1* rs3743527, rs212091, and s212090 and *CBR1* rs9024) were associated with chemotherapy toxicity. A future prospective study with a larger patient population will confirm the current data.

We found that *NT5C2* rs10786736 and rs8139 were independent predictors for AML survival. The rs10786736 GG genotype compared with the CG genotype has a shorter OS and RFS, and the rs8139 TT+CT genotypes have longer OS and RFS than the CC genotype. Cytosolic 5′-nucleotidase II (NT5C2) functions in dephosphorylating nucleoside triphosphates and is able to deactivate Ara-C through the de-phosphorylation of Ara-C monophosphate to Ara-C, and an increase in NT5C2 expression has shown to associate with the development of resistance to nucleoside analog-based chemotherapies, such as Ara-C, gemcitabine, and cladribine, in different cancer cell lines [18, 19]. Levels of NT5C2 expression correlated with the resistance of primary leukemic cells to Ara-C treatment in vitro and predicted a poorer clinical outcome in AML patients [20, 21]. However, to date, there are only a few of studies reporting the association between *NT5C2*SNPs and outcomes in AML patients. For example, Mitra AK [6] sequenced *NT5C2* and identified 41 genetic variants with twenty-five novel SNPs and then associated *NT5C2*SNPs with *NT5C2* mRNA levels and Ara-C sensitivity in HapMap cancer cell lines as well as with treatment responses of pediatric AML patients to Ara-C. To date, this is the only study of rs10786736 with an outcome showing no statistical association between rs10786736 and Ara-CTP levels (after 1 and 2 days of treatment) and the clinical response of AML patients. However, the GG genotype was associated with higher NT5C2 mRNA levels and Ara-C cytotoxicity as well as tumor cell resistance to Ara-C in HapMap samples, which was also associated with Ara-C sensitivity of primary AML leukemic blasts. In consensus with the HapMap results, the GG genotype was associated with a greater Ara-C LC50 value versus the CG genotype [6]. This finding supported our current data on *NT5C2* variant association with AML CR and survival in AML patients.

In the current study, we also observed *SLC29A1* rs3734703 to associate with CR and survival in AML patients who received Ara-C based treatment. The solute carrier family 29 (also known as the equilibrative nucleoside transporter), member 1 (SLC29A1, or ENT1) plays a crucial role in cell uptake of anticancer nucleoside agents or nucleosides from the surrounding medium, and SLC29A1 is known to transport approximately 80% of Ara-C into leukemic cells [22, 23]. In mechanistic studies of different Ara-C resistances in leukemic cells, altered SLC29A1 expression was associated with treatment resistance [24]. Many studies also reported that functional abnormalities in SLC29A1were associated with AML resistance to Ara-C [25, 26]. In previous studies, *SLC29A1* genetic variants (such as rs693955, rs9394992, and rs324148) were associated with treatment outcomes in AML patients [27, 28]. In terms of rs3734703, only two studies reported that AML patients with a high frequency of the major “C” allele (in other words, low MAF) of rs3734703 had a poor response to Ara-C-based therapy [29], while another study [30] did not find any rs3734703 associations with CR or RFS after induction chemotherapy with AML. However, the combination of rs3734703 AA or AC genotype with the *TYMS* rs2612100 AA genotype was significantly associated with shorter RFS compared to the wild type, which is in line with our current data. A possible explanation of these results is that *SLC29A1* rs3734703 may increase SLC29A1 expression and in turn induce the uptake of Ara-C by AML cells and increase tumor cell apoptosis and clinical CR.

Furthermore, ribonucleotide reductase (RR) is composed of dimerized large (RRM1) and small (RRM2) subunits and regulates intracellular pools of deoxy-CTP (dCTP), the expression of which leads to tumor cell resistance to nucleoside analogs, such as Ara-C treatment [31]. Indeed, tumor cell lines and leukemic blasts with high dCTP expression levels were resistant to Ara-C [32–34]. The biochemical modulation of Ara-C by nucleoside analogs, such as fludarabine and cladribine, stimulated Ara-CTP accumulation in leukemic cells from adult and pediatric patients [35, 36]. Our current study showed that *RRM1* SNP rs1042919 was associated with chemotherapy response of Chinese AML patients to AraC-based chemotherapy and further supported data from a previous study [37]. The study [37] identified *RRM1* and *RRM2* genetic variations by sequencing of the genomic DNA from HapMap European and African ancestry panels and revealed that the *RRM1* rs1042919 AT genotype was associated with lower intracellular Ara-CTP levels in leukemic cells after 1 day of treatment with Ara-C alone and 2 day treatment with Ara-C in combination with cladribine. The same research team also observed an association between *RRM1* rs1042919 and poor survival and the risk of relapse in patients with AML97 treatment [38]. However, in the current study, we did not observe any associations between *RRM1* rs1042919 and survival in AML patients. One of the reasons for this finding may be because patients in the AML97 study received cladribine in combination with Ara-C, whereas our patients only received Ara-C-based therapy. Cladribine, as a purine analog, mimics the nucleoside adenosine to inhibit the activity of adenosine deaminase, which reduces the cellular pool of deoxynucleotide levels [39].

Our current study also assessed *DCTD* rs7278 polymorphisms in association with the treatment response of AML patients to AraC-based chemotherapy and found that TT/TC genotypes reached a higher CR rate compared to the wild genotype. DCTD enzyme can deaminate Ara-CMP to Ara-UMP; however, its role in Ara-C-resistance is poorly understood. Previous studies suggested a substantial role for DCTD in Ara-CMP metabolism in T-lymphoblastic leukemia [32, 40, 41], and the association between *DCTD* polymorphisms and clinical outcome warrants further investigation [40, 41].

Since the 1960s, anthracyclines have been widely prescribed for AML treatment, and polymorphisms of anthracyclines-metabolic pathway genes have also been widely studied [43–46]. Association of anthracyclines disposition in blast cells and different tissues with treatment outcomes of AML patients could be influenced by SNPs of the anthracyclines pathway genes (such as CBR and NQO1) and the efflux transporters (ABCB1 and ABCC1) [16]. The current study failed to show any significant associations between the poly-miRTS of anthracycline-pathway genes and CR or OS/RFS of AML; however, we did show that four poly-miRTS were associated with chemotherapy toxicity, i.e., ABCC1 rs3743527, rs212091, and rs212090 were significantly associated with myelosuppression and gastrointestinal reaction, which were reported to associate with a risk in developing lung cancer [42]. Moreover, cardiotoxicity was the most common anthracycline-induced toxicity [43, 44]; however, the current study only observed one poly-miRTS (*CBR* rs9024) to be significantly associated with cardiotoxicity. The *CBR* rs9024 GG genotype was associated with an increase in the clearance and reduction of doxorubicin exposure levels in Asian breast cancer patients [45]. However, another study did not show the GA/AA genotype to increase the risk of cardiomyopathy in childhood cancers (including 68 AML) [46]. Kalabus [47] reported that *CBR*rs9024 impacted *CBR1* phenotypes in the liver, and the homozygosis of the major G allele was associated with significantly higher CBR1 protein levels and CBR doxorubicin reductase activity. The same research team also carried out a functional study [48] and showed that hsa-miR-574-5p and hsa-miR-921 significantly decreased the luciferase activity of CHO cells after transfection with the *CBR1* 3′-UTR construct carrying the major rs9024 G allele (35 and 46%, respectively), as well as a decreased level of CBR1 protein (48 and 40%, respectively) and CBR1 activity (54 and 18%, respectively) in lymphoblastoid cells that contain the homozygous major rs9024 G allele. On this basis, we speculate that the patients with *CBR* rs9024 GG may have a lower risk in developing cardiotoxicity after anthracycline treatment, and our current data supported this notion.

Last but not least, platelet counts at diagnosis were also an independent predictor for CR of AML patients after Ara-C based chemotherapy; these data are novel and supported previous studies of cytogenetic and molecular abnormalities as independent predictors for the prognosis of AML patients [49–51]. Moreover, our current study showed that adverse cytogenetic abnormalities only counted for 25.7% (n = 53) of patients and that the majority of patients were in the intermediate and favorable risk stratifications without a difference in survival. Hence, only cytogenetic alterations may not be sufficient to predict AML prognosis, and our current results indicate that poly-miRTS in genes involved in the metabolic pathways of Ara-C and may be useful biomarkers to predict treatment response and prognosis in AML. However, future functional studies are required to define the role of these SNPs in AML. There are some limitations of our current study; for example, our current study is retrospective and patients showed some differences in treatment regimens, and the study population is relatively small.

## Conclusions

Although further functional evaluations and confirmatory studies are need to further support our current data, the current study demonstrated that the miRNA-binding site SNPs of Ara-C and anthracycline metabolic pathways genes could predict treatment responses, side effects and survival in AML patients after Ara-C-based chemotherapy. They could provide insightful information to identify patients with an increased risk of adverse reactions or decreased chemotherapy responses for future individualized chemotherapy and predicting outcomes in AML patients.

## Additional files

**Additional file 1: Table S1.** Primer sequences used to genotype 17 poly-miRTS with the MassARRAY platform.

**Additional file 2: Table S2.** Associations between clinical characteristics and chemotherapy toxicity.

**Additional file 3: Table S3.** Associations between clinical characteristics and chemotherapy response and disease survival.

**Additional file 4: Table S4.** Genotype distribution and response to chemotherapy of 17 poly-miRTS.

**Additional file 5: Table S5.** Genotype distribution of 17 poly-miRTS between the characteristics of AML patients.

**Additional file 6: Table S6.** Associations between 17 poly-miRTSs and adverse events after chemotherapy in AML patients (Chi square/Fisher’s exact test).

**Additional file 7: Figure S1.** Kaplan–Meier evaluation of associations between *NT5C2* rs12573199, *DCTD* rs7278, rs3811810, rs851 and rs9542, *SLC28A1* rs8025045, and *RRM1* rs1042919 polymorphisms with OS in AML patients. **Figure S2.** Kaplan–Meier evaluation of associations between *NT5C2* rs12573199, *DCTD* rs7278, rs3811810, rs851and rs9542, *SLC28A1* rs8025045, and *RRM1* rs1042919 polymorphisms with RFS in AML patients. **Figure S3.** Kaplan–Meier evaluation of associations between rs9024, rs3842, rs212090, rs212091, rs3743527, rs4148380, rs10517 polymorphisms with OS in AML patients. **Figure S4.** Kaplan–Meier evaluation of associations between rs9024, rs3842, rs212090, rs212091, rs3743527, rs4148380, and rs10517 polymorphisms with RFS in AML patients.

**Additional file 8: Table S7.** Combined effects of rs10786736, rs3734703 and rs8139 genotypes on survival of AML patients.

## Abbreviations

Ara-C: cytarabine
poly-miRTS: polymorphisms at microRNA-binding sites
AML: acute myeloid leukemia
SNPs: single nucleotide polymorphisms
CR: complete remission
OS: overall survival
RFS: relapse-free survival
OR: odds ratio
HR: hazard ratio
CI: confidence intervals
*DCK*: deoxycytidine kinase
*CDA*: cytidine deaminase
*SLC29A1*: solute carrier family 29 member 1
*SLC28A3, SLC28A1*: solute carrier family 28 member
*NT5C3*: 5′ nucleotidase 3
*NT5C2*: 5′ nucleotidase 2
*DCTD*: deoxycytidylate deaminase
*RRM1* and *RRM2*: ribonucleotide reductase
*ABCA3*, *ABCB1*, *ABCC1*, *ABCG2, ABCC10, ABCC11*: 10 anthracycline-associated genes ATP binding cassette subfamily A, B, C, G member
*GSTM1, GSTT1*: glutathione S-transferase
C*BR3* and *CBR1*: carbonyl reductases
