# Supplementary material for: Polymorphisms at microRNA binding sites of Ara-C and anthracyclines-metabolic pathway genes are associated with outcome of acute myeloid leukemia patients
Source: J Transl Med. 2017 Nov 15;15:235. doi: 10.1186/s12967-017-1339-9 (PMC5688732; doi:10.1186/s12967-017-1339-9)
Supplement: Supplementary file 1 — Additional file 1: Table S1. Primer sequences used to genotype 17 poly-miRTS with the MassARRAY platform. [file 12967_2017_1339_MOESM1_ESM.docx]

Table S1 Primer sequences used to genotype 17 poly-miRTS with the MassARRAY platform

| **SNP ID** | **Forward primers** | **Reverse primers** | **Extension primers** |
| --- | --- | --- | --- |
| rs3734703 | ACGTTGGATGATGATGGGTAAGAACTGGGC | ACGTTGGATGTATACTCCATTCTCCCCTGC | TCTGTGTTCTCTCCATGT |
| rs10786736 | ACGTTGGATGGCATGTATATGGCTCCACTG | ACGTTGGATGTCCACTCTGAGACTCTTTCC | TTTTCCTCGTGTATCCA |
| rs8139 | ACGTTGGATGCCCTAACATACACAGTAATG | ACGTTGGATGAAAATCCCAATCCCAAGTCG | CACAATCCCAAGTCGAAATCAC |
| rs12573199 | ACGTTGGATGCCTCAAAATAGAATCCTGCC | ACGTTGGATGCAAGGCTGCTTCTCTATCAC | GCGTTGCACAGAAGTATGCTCTACT |
| rs3811810 | ACGTTGGATGACTGCTACATGTGCTGGAAG | ACGTTGGATGTTCACTTGGGCCAGATGTTC | TCAGATCAGTGTGTCTGCT |
| rs7278 | ACGTTGGATGCTCCATGCTTTCACCTACAC | ACGTTGGATGTTAAAGAGAAGGGGCACAGC | CCAGCCACACGCTCCCC |
| *rs851* | ACGTTGGATGCCCCAACATGGATGTGTTGC | ACGTTGGATGTTTCTCTCCAGTTGCAGAGG | GGGCCTTGTACATGAAC |
| rs9542 | ACGTTGGATGACTCTCGTTCTAGCCCTGTG | ACGTTGGATGCTCTCTTGTCACATGGAATC | AGGTGTTTGAACTATTGCTTTAGG |
| rs1042919 | ACGTTGGATGGGTGAAAATCCCAATATATG | ACGTTGGATGCCTGTTGCAGGCAAAAGGAG | CAATTGATTTAAAGTACTGTTAATGATG |
| rs8025045 | ACGTTGGATGACTTAGACCCAGCTCAATCC | ACGTTGGATGCCTTCCTTATGTCCTCACTC | TCTTCACACTCACTCCATGA |
| rs9024 | ACGTTGGATGGATCTCTTATCAATTAGCAC | ACGTTGGATGGTAGTCAACTGAGTGCGTAG | TAGGTTGCTCAATTAGTAGTA |
| rs3842 | ACGTTGGATGTAAAATCTACTTTAATTCTG | ACGTTGGATGGGAACAGAGTGAGAGACATC | GAGCATCAAGTGGAGAGAAATC |
| rs212090 | ACGTTGGATGCTCCTTAATATTTACCCCAC | ACGTTGGATGTCCAGGCTTTCCCTTTTTTC | AACAATCAATGCTGTTATTACTG |
| rs212091 | ACGTTGGATGGTTCCTGGAAGAAAACAGGG | ACGTTGGATGTTCCACTTTGGGCTCTAACG | TCTCTAACGAGACACCTTA |
| rs3743527 | ACGTTGGATGTGCAGTTTTGTGGTTGAGGG | ACGTTGGATGTTGGTAAGAGGACCATCCAG | TATGGGACACTGCCAAG |
| rs4148380 | ACGTTGGATGTTAGTTACTGATGCTCTTCC | ACGTTGGATGTGTTTTTCTCCCCGGAACAG | AGGGGTATTCAAAGATGGGTTCTTTT |
| rs10517 | ACGTTGGATGTACAATTGTACCCCAAGGTC | ACGTTGGATGCTTTATCATTGCTAAACTG | TTGCTAAACTGATGACTTAC |
