# Supplementary material for: Polymorphisms at microRNA binding sites of Ara-C and anthracyclines-metabolic pathway genes are associated with outcome of acute myeloid leukemia patients
Source: J Transl Med. 2017 Nov 15;15:235. doi: 10.1186/s12967-017-1339-9 (PMC5688732; doi:10.1186/s12967-017-1339-9)
Supplement: Supplementary file 2 — Additional file 2: Table S2. Associations between clinical characteristics and chemotherapy toxicity. [file 12967_2017_1339_MOESM2_ESM.docx]

Table S2. Associations of clinical Characteristics with chemotherapy toxicity

| **Characteristics** | Total | Myelosuppression | | Gastrointestinal reaction reaction | | Liver function damage | | Cardiotoxicity | |
| --- | --- | --- | --- | --- | --- | --- | --- | --- | --- |
|  | N | N | *P* | N | *P* | N | *P* | N | *P* |
| **Sex** |  |  | 0.504 |  | 0.685 |  | 0.314 |  | 0.807 |
| Female | 105 | 87 |  | 35 |  | 14 |  | 6 |  |
| Male | 101 | 80 |  | 31 |  | 9 |  | 5 |  |
| **Risk stratifications** |  |  | 0.057 |  | 0.766 |  | **0.009** |  | **0.004** |
| Low | 35 | 31 |  | 11 |  | 8 |  | 6 |  |
| Intermediate | 118 | 89 |  | 40 |  | 7 |  | 3 |  |
| High | 53 | 47 |  | 15 |  | 8 |  | 2 |  |
| **BM blasts, %** |  |  | 0.101 |  | 0.475 |  | 0.633 |  | 0.125 |
| ≥67.2 | 100 | 85 |  | 34 |  | 10 |  | 8 |  |
| ＜67.2 | 99 | 75 |  | 29 |  | 12 |  | 3 |  |
| **Age, year** |  |  | 0.079 |  | 0.542 |  | 0.606 |  | 0.833 |
| ≥43 | 106 | 81 |  | 36 |  | 13 |  | 6 |  |
| ＜43 | 100 | 86 |  | 30 |  | 10 |  | 5 |  |
| **WBC, ×109/L** |  |  | 0.806 |  | 0.839 |  | 0.248 |  | 0.113 |
| ≥20.7 | 104 | 85 |  | 34 |  | 9 |  | 3 |  |
| ＜20.7 | 102 | 82 |  | 32 |  | 14 |  | 8 |  |
| **HB, g/L** |  |  | 0.411 |  | 0.397 |  | 0.475 |  | 1.000 |
| ≥81 | 103 | 81 |  | 30 |  | 10 |  | 5 |  |
| ＜81 | 101 | 84 |  | 35 |  | 13 |  | 5 |  |
| **Plt, ×10^9^L** |  |  | 0.859 |  | 0.232 |  | 0.507 |  | 0.353 |
| ＜38.5 | 103 | 83 |  | 29 |  | 10 |  | 4 |  |
| ≥38.5 | 103 | 84 |  | 37 |  | 13 |  | 7 |  |
| **FAB** |  |  | 0.469 |  | 0.389 |  | 0.174 |  | 0.515 |
| M0 | 2 | 1 |  | 1 |  | 0 |  | 0 |  |
| M1 | 14 | 12 |  | 3 |  | 3 |  | 2 |  |
| M2 | 104 | 84 |  | 40 |  | 12 |  | 7 |  |
| M4 | 31 | 28 |  | 8 |  | 0 |  | 1 |  |
| M5 | 46 | 34 |  | 11 |  | 7 |  | 1 |  |
| M6 | 7 | 6 |  | 3 |  | 1 |  | 0 |  |
| M7 | 2 | 2 |  | 0 |  | 0 |  | 0 |  |

Abbreviations: FAB, French–American–British classification; WBC, White blood cells; BM, bone marrow; Plt, platelets；HB, hemoglobin; *P* values were calculated by Chi-square test or Fish exact test
