# Supplementary material for: Polymorphisms at microRNA binding sites of Ara-C and anthracyclines-metabolic pathway genes are associated with outcome of acute myeloid leukemia patients
Source: J Transl Med. 2017 Nov 15;15:235. doi: 10.1186/s12967-017-1339-9 (PMC5688732; doi:10.1186/s12967-017-1339-9)
Supplement: Supplementary file 3 — Additional file 3: Table S3. Associations between clinical characteristics and chemotherapy response and disease survival. [file 12967_2017_1339_MOESM3_ESM.docx]

| **ccharacteristicsss** | Total, n | CR, n | *P* | OS | *P* | RFS | *P* |
| --- | --- | --- | --- | --- | --- | --- | --- |
| **Sex** |  |  | 0.284 |  | 0.130 |  | 0.659 |
| Female Female | 105 | 87 |  | 1506±100 |  | 1128±106 |  |
| Male | 101 | 89 |  | 1273±108 |  | 1061±102 |  |
| **Risk stratifications** |  |  | 0.088 |  | 0.022 |  | 0.008 |
| Low risk | 35 | 34 |  |  |  | 1532±151 |  |
| Intermediate risk | 118 | 99 |  | 1473±82* |  | 1096±99 |  |
| High risk | 53 | 43 |  | 1048±113 |  | 740±105 |  |
| **BM blasts, %** |  |  | 0.411 |  | 0.683 |  | 0.782 |
| ≥67.2 | 100 | 87 |  | 1440±107 |  | 1066±119 |  |
| ＜67.2 | 99 | 82 |  | 1382±96 |  | 1136±100 |  |
| **Age, year** |  |  | 0.311 |  | 0.334 |  | 0.283 |
| ≥43 | 106 | 88 |  | 1460±89 |  | 1170±94 |  |
| ＜43 | 100 | 88 |  | 1281±143 |  | 985±143 |  |
| **WBC, ×109/L** |  |  | 0.259 |  | 0.550 |  | 0.674 |
| ≥20.7 | 104 | 86 |  | 1421±108 |  | 1110±112 |  |
| ＜20.7 | 102 | 90 |  | 1378±101 |  | 1103±104 |  |
| **Hemoglobin, g/L** |  |  | 0.259 |  | 0.252 |  | 0.791 |
| ≥81 | 105 | 87 |  | 1495±98 |  | 1143±108 |  |
| ＜81 | 101 | 89 |  | 1345±108 |  | 1115±108 |  |
| **Platelets, ×109/L** |  |  | 0.006 |  | 0.057 |  | 0.936 |
| ＜38.5 | 103 | 81 |  | 1290±111 |  | 1153±114 |  |
| ≥38.5 | 103 | 95 |  | 1525±96 |  | 1083±102 |  |
| **FAB classification** |  |  | 0.213 |  | 0.811 |  | 0.150 |
| M0 | 2 | 1 |  |  |  |  |  |
| M1 | 14 | 14 |  |  |  |  |  |
| M2 | 104 | 87 |  |  |  |  |  |
| M4 | 31 | 27 |  |  |  |  |  |
| M5 | 46 | 39 |  |  |  |  |  |
| M6 | 7 | 7 |  |  |  |  |  |
| M7 | 2 | 1 |  |  |  |  |  |

**Table S3. Associations of clinical characteristics with chemotherapy response and disease survival**

* low+ intermediate risk (because there were no mortality in low risk group)
