# Supplementary material for: Polymorphisms at microRNA binding sites of Ara-C and anthracyclines-metabolic pathway genes are associated with outcome of acute myeloid leukemia patients
Source: J Transl Med. 2017 Nov 15;15:235. doi: 10.1186/s12967-017-1339-9 (PMC5688732; doi:10.1186/s12967-017-1339-9)
Supplement: Supplementary file 4 — Additional file 4: Table S4. Genotype distribution and response to chemotherapy of 17 poly-miRTS. [file 12967_2017_1339_MOESM4_ESM.docx]

**Table S4. Genotype distribution and response to chemotherapy of 17 poly-miRTS**

| SNP genotype | Total, n | | Allele frequencies | | *P^a^* | | CR, n (%) | | Non-CR | *P** |  |
| --- | --- | --- | --- | --- | --- | --- | --- | --- | --- | --- | --- |
| rs3734703 | 206 | | C | A | 0.082 | |  | |  |  |  |
| CC | 122 | | 0.75 | 0.25 |  | | 108（88.5） | | 14 |  |  |
| CA | 67 | |  |  |  | | 52（77.6） | | 15 | 0.046 |  |
| AA | 17 | |  |  |  | | 16（94.1） | | 1 | 0.780 |  |
| ^c^CC+AA | 139 | |  |  |  | | 124（89.2） | | 15 | 0.027 |  |
| rs10786736 | 206 | | C | G | 0.191 | |  | |  |  |  |
| GG | 96 | | 0.30 | 0.70 |  | | 81（84.4） | | 15 |  |  |
| CG | 95 | |  |  |  | | 81（85.3） | | 14 | 0.864 |  |
| CC | 15 | |  |  |  | | 14（93.3） | | 1 | 0.601 |  |
| rs8139 | 206 | | T | C | 0.086 | |  | |  |  |  |
| TT | 51 | | 0.53 | 0.47 |  | | 41（80.4） | | 10 |  |  |
| CT | 115 | |  |  |  | | 103（89.6） | | 12 | 0.108 |  |
| CC | 40 | |  |  |  | | 32（80.0） | | 8 | 0.963 |  |
| rs12573199 | 206 | | A | T | 0.094 | |  | |  |  |  |
| AA | 163 | | 0.90 | 0.10 |  | | 141（86.5） | | 22 |  |  |
| TA | 43 | |  |  |  | | 35（81.4） | | 8 | 0.398 |  |
| TT | 0 | |  |  |  | |  | |  |  |  |
| \| rs3811810 \| \| --- \| | 206 | | G | A | 0.513 | |  | |  |  |  |
| GG | 167 | | 0.89 | 0.11 |  | | 138（82.6） | | 29 |  |  |
| GA | 36 | |  |  |  | | 35（97.2） | | 1 | 0.025 |  |
| AA | 3 | |  |  |  | | 3（100.0） | | 0 | 1.000 |  |
| ^b^AA+GA | 39 | |  |  |  | | 38（97.4） | | 1 | 0.018 |  |
| rs7278 | 206 | | T | C | 0.269 | |  | |  |  |  |
| CC | 165 | | 0.11 | 0.89 |  | | 136（82.4） | | 29 |  |  |
| TC | 37 | |  |  |  | | 36（97.3） | | 1 | 0.021 |  |
| TT | 4 | |  |  |  | | 4（100.0） | | 0 | 1.000 |  |
| ^b^TT+TC | 41 | |  |  |  | | 40（97.6） | | 1 | 0.014 |  |
| rs9542 | 206 | | G | A | 0.668 | |  | |  |  |  |
| GG | 28 | | 0.36 | 0.64 |  | | 25（89.3） | | 3 |  |  |
| AG | 92 | |  |  |  | | 75（81.5） | | 17 | 0.499 |  |
| AA | 86 | |  |  |  | | 76（88.4） | | 10 | 1.000 |  |
| rs8025045 | 206 | | T | G | 0.768 | |  | |  |  |  |
| GG | 182 | | 0.06 | 0.94 |  | | 154（84.6） | | 28 |  |  |
| GT | 23 | |  |  |  | | 22（95.7） | | 1 | 0.265 |  |
| TT | 1 | |  |  |  | | 0（0） | | 1 | 0.158 |  |
| rs1042919 | 206 | | A | T | 0.901 | |  | |  |  |  |
| TT | 109 | | 0.26 | 0.74 |  | | 101（92.7） | | 8 |  |  |
| AT | 88 | |  |  |  | | 68（77.3） | | 20 | 0.002 |  |
| AA | 9 | |  |  |  | | 7（77.8） | | 2 | 0.169 |  |
| ^b^AA+AT | 97 | |  |  |  | | 75（77.3） | | 22 | 0.002 |  |
| rs851 | 206 | | A | G | 0.542 | |  | |  |  |  |
| GG | 14 | | 0.75 | 0.25 |  | | 11（78.6） | | 3 |  |  |
| GA | 73 | |  |  |  | | 57（78.1） | | 16 | 1.000 |  |
| AA | 119 | |  |  |  | | 108（90.8） | | 11 | 0.345 |  |
| rs3842 | 206 | G | | A | 0.552 |  | |  | |  | |
| AA | 116 | 0.25 | | 0.75 |  | 99（85.3） | | 17 | |  | |
| AG | 75 |  | |  |  | 64（85.3） | | 11 | | 0.998 | |
| GG | 15 |  | |  |  | 13（86.7） | | 2 | | 1.000 | |
| rs4148380 | 206 | A | | G | 0.354 |  | |  | |  | |
| GG | 181 | 0.06 | | 0.94 |  | 156（86.2） | | 25 | |  | |
| GA | 25 |  | |  |  | 20（80.0） | | 5 | | 0.603 | |
| rs3743527 | 206 | T | | C | 0.327 |  | |  | |  | |
| CC | 63 | 0.43 | | 0.57 |  | 54（85.7） | | 9 | |  | |
| TC | 108 |  | |  |  | 92（85.2） | | 16 | | 0.925 | |
| TT | 35 |  | |  |  | 30（85.7） | | 5 | | 1.000 | |
| rs212091 | 206 | A | | G | 0.751 |  | |  | |  | |
| AA | 122 | 0.77 | | 0.23 |  | 107（87.7） | | 15 | |  | |
| AG | 72 |  | |  |  | 58（80.6） | | 14 | | 0.177 | |
| GG | 12 |  | |  |  | 11（91.7） | | 1 | | 1.000 | |
| rs212090 | 206 | A | | T | 0.848 |  | |  | |  | |
| TT | 135 | 0.19 | | 0.81 |  | 116（85.9） | | 19 | |  | |
| AT | 63 |  | |  |  | 53（84.1） | | 10 | | 0.739 | |
| AA | 8 |  | |  |  | 7（87.5） | | 1 | | 1.000 | |
| rs10517 | 206 | T | | C | 0.431 |  | |  | |  | |
| CC | 92 | 0.33 | | 0.67 |  | 83（90.2） | | 9 | |  | |
| CT | 95 |  | |  |  | 79（83.2） | | 16 | | 0.156 | |
| TT | 19 |  | |  |  | 14（73.7） | | 5 | | 0.110 | |
| rs9024 | 206 | A | | G | 0.318 |  | |  | |  | |
| GG | 123 | 0.24 | | 0.76 |  | 102（82.9） | | 21 | |  | |
| GA | 69 |  | |  |  | 62（89.9） | | 7 | | 0.192 | |
| AA | 14 |  | |  |  | 12（85.7） | | 2 | | 1.000 | |

^a^Hardy-Weinberg equilibrium analysis; P*calculated by Chi-square test or Continuity Correction Chi-Square test or Fish exact test; ^b^dominant model; **^c^**Overdominance model;
