# Supplementary material for: Polymorphisms at microRNA binding sites of Ara-C and anthracyclines-metabolic pathway genes are associated with outcome of acute myeloid leukemia patients
Source: J Transl Med. 2017 Nov 15;15:235. doi: 10.1186/s12967-017-1339-9 (PMC5688732; doi:10.1186/s12967-017-1339-9)
Supplement: Supplementary file 5 — Additional file 5: Table S5. Genotype distribution of 17 poly-miRTS between the characteristics of AML patients. [file 12967_2017_1339_MOESM5_ESM.docx]

**Table S5.** Genotype distribution of 17 poly-miRTS between the characteristics of AML patients

| SNP genotype | Total, n | Chemotherapy regimens# | | | | | *P** |
| --- | --- | --- | --- | --- | --- | --- | --- |
|  |  | DA | IA | MA | TA | CAG |  |
| rs3734703 |  |  |  |  |  |  | 0.164 |
| CC | 122 | 51 | 42 | 4 | 17 | 8 |  |
| AA | 17 | 8 | 5 | 1 | 3 | 0 |  |
| CA | 67 | 30 | 14 | 5 | 7 | 11 |  |
| rs10786736 |  |  |  |  |  |  | 0.689 |
| GG | 96 | 42 | 29 | 3 | 10 | 12 |  |
| CC | 15 | 5 | 5 | 1 | 3 | 1 |  |
| CG | 95 | 42 | 27 | 6 | 14 | 6 |  |
| rs8139 |  |  |  |  |  |  |  |
| TT | 51 | 27 | 12 | 3 | 6 | 3 | 0.664 |
| CC | 40 | 16 | 13 | 2 | 3 | 6 |  |
| CT | 115 | 46 | 36 | 5 | 18 | 10 |  |
| rs12573199 |  |  |  |  |  |  | 0.923 |
| AA | 163 | 69 | 50 | 7 | 22 | 15 |  |
| TA | 43 | 20 | 11 | 3 | 5 | 4 |  |
| TT | 0 |  |  |  |  |  |  |
| \| rs3811810 \| \| --- \| |  |  |  |  |  |  | 0.698 |
| GG | 167 | 75 | 46 | 8 | 22 | 16 |  |
| AA | 3 | 1 | 1 | 0 | 0 | 1 |  |
| GA | 36 | 13 | 14 | 2 | 5 | 2 |  |
| rs7278 |  |  |  |  |  |  | 0.835 |
| CC | 165 | 71 | 48 | 7 | 23 | 16 |  |
| TT | 4 | 1 | 2 | 0 | 1 | 0 |  |
| TC | 37 | 17 | 11 | 3 | 3 | 3 |  |
| rs9542 |  |  |  |  |  |  | 0.963 |
| AA | 86 | 36 | 25 | 4 | 11 | 10 |  |
| GG | 28 | 6 | 6 | 2 | 4 | 2 |  |
| AG | 92 | 39 | 30 | 4 | 12 | 7 |  |
| rs8025045 |  |  |  |  |  |  | 0.461 |
| TT | 1 | 1 | 0 | 0 | 0 | 0 |  |
| GG | 182 | 74 | 57 | 10 | 23 | 18 |  |
| GT | 23 | 14 | 4 | 0 | 4 | 1 |  |
| rs1042919 |  |  |  |  |  |  | 0.082 |
| TT | 109 | 41 | 40 | 5 | 12 | 11 |  |
| AA | 9 | 7 | 0 | 1 | 0 | 1 |  |
| AT | 88 | 41 | 21 | 4 | 15 | 7 |  |
| rs851 |  |  |  |  |  |  | 0.865 |
| AA | 119 | 51 | 37 | 6 | 13 | 12 |  |
| GG | 14 | 8 | 2 | 1 | 2 | 1 |  |
| GA | 73 | 30 | 22 | 3 | 12 | 6 |  |
| rs3842 |  |  |  |  |  |  |  |
| GG | 15 | 6 | 4 | 0 | 3 | 2 |  |
| AA | 116 | 53 | 34 | 5 | 15 | 9 |  |
| AG | 75 | 30 | 23 | 5 | 9 | 8 |  |
| rs4148380 |  |  |  |  |  |  | 0.975 |
| GG | 181 | 79 | 53 | 9 | 24 | 16 |  |
| GA | 25 | 10 | 8 | 1 | 3 | 3 |  |
| rs3743527 |  |  |  |  |  |  | 0.780 |
| CC | 63 | 30 | 16 | 3 | 10 | 4 |  |
| TT | 35 | 16 | 13 | 1 | 3 | 2 |  |
| TC | 108 | 43 | 32 | 6 | 14 | 13 |  |
| rs212091 |  |  |  |  |  |  | 0.090 |
| GG | 12 | 2 | 6 | 1 | 3 | 0 |  |
| AA | 122 | 53 | 38 | 4 | 18 | 9 |  |
| AG | 72 | 34 | 17 | 5 | 6 | 10 |  |
| rs212090 |  |  |  |  |  |  | 0.524 |
| TT | 135 | 58 | 42 | 7 | 14 | 14 |  |
| AA | 8 | 2 | 3 | 1 | 2 | 0 |  |
| AT | 63 | 29 | 16 | 2 | 11 | 5 |  |
| rs10517 |  |  |  |  |  |  | 0.250 |
| TT | 19 | 9 | 4 | 1 | 2 | 3 |  |
| CC | 92 | 39 | 28 | 8 | 12 | 5 |  |
| CT | 95 | 41 | 29 | 1 | 13 | 11 |  |
| rs9024 |  |  |  |  |  |  | 0.766 |
| AA | 14 | 5 | 6 | 0 | 2 | 1 |  |
| GG | 123 | 49 | 39 | 8 | 16 | 11 |  |
| GA | 69 | 35 | 16 | 2 | 9 | 7 |  |
| Risk stratifications |  |  |  |  |  |  | 0.190 |
| Low | 35 | 11 | 15 | 0 | 6 | 3 |  |
| Intermediate | 118 | 59 | 29 | 5 | 14 | 11 |  |
| High | 53 | 19 | 17 | 5 | 7 | 5 |  |

# The first course induction chemotherapy regimens；P*calculated by Chi-square test.
