# Supplementary material for: Polymorphisms at microRNA binding sites of Ara-C and anthracyclines-metabolic pathway genes are associated with outcome of acute myeloid leukemia patients
Source: J Transl Med. 2017 Nov 15;15:235. doi: 10.1186/s12967-017-1339-9 (PMC5688732; doi:10.1186/s12967-017-1339-9)
Supplement: Supplementary file 6 — Additional file 6: Table S6. Associations between 17 poly-miRTSs and adverse events after chemotherapy in AML patients (Chi square/Fisher’s exact test). [file 12967_2017_1339_MOESM6_ESM.docx]

**Table S6. Associations of 17 poly-miRTSs with adverse events after chemotherapy in AML patients (Chi-square/Fisher’s exact test)**

| Gene genotype | Tota, n | Gastrointestinal reaction, n | | | | Liver function damage, n | | | | Myelosuppression,n | | | | Cardiotoxicity, n | | | | |
| --- | --- | --- | --- | --- | --- | --- | --- | --- | --- | --- | --- | --- | --- | --- | --- | --- | --- | --- |
|  |  | Y | N | *P* | | Y | N | | *P* | Y | N | *P* | | Y | N | | *P* | |
| rs3734703 | 206 | 66 |  | 0.735 | | 23 |  | | 0.974 | 167 |  | 0.708 | | 11 |  | | 0.243 | |
| CC | 122 | 40 | 82 |  | | 14 | 108 | |  | 98 | 24 |  | | 7 | 115 | |  | |
| CA | 67 | 22 | 45 |  | | 7 | 60 | |  | 54 | 13 |  | | 2 | 65 | |  | |
| AA | 17 | 4 | 13 |  | | 2 | 15 | |  | 15 | 2 |  | | 2 | 15 | |  | |
| rs10786736 | 206 | 66 |  | 0.211 | | 23 |  | | 0.477 | 167 |  | 0.827 | | 11 |  | | 0.206 | |
| GG | 96 | 25 | 71 |  | | 8 | 88 | |  | 77 | 19 |  | | 3 | 93 | |  | |
| CG | 95 | 36 | 59 |  | | 13 | 82 | |  | 77 | 18 |  | | 8 | 87 | |  | |
| CC | 15 | 5 | 10 |  | | 2 | 13 | |  | 13 | 2 |  | | 0 | 15 | |  | |
| re8139 | 206 | 66 |  | 0.750 | | 23 |  | | 0.317 | 167 |  | 0.163 | | 11 |  | | 0.056 | |
| TT | 51 | 16 | 35 |  | | 6 | 45 | |  | 45 | 6 |  | | 1 | 50 | |  | |
| CT | 115 | 39 | 76 |  | | 15 | 100 | |  | 93 | 22 |  | | 10 | 105 | |  | |
| CC | 40 | 11 | 29 |  | | 2 | 38 | |  | 29 | 11 |  | | 0 | 40 | |  | |
| rs12573199 | 206 | 66 |  | 0.775 | | 23 |  | | 0.275 | 167 |  | 0.169 | | 11 |  | | 0.465 | |
| AA | 163 | 53 | 110 |  | | 16 | 147 | |  | 129 | 34 |  | | 10 | 153 | |  | |
| TA | 43 | 13 | 30 |  | | 7 | 36 | |  | 38 | 5 |  | | 1 | 42 | |  | |
| \| rs3811810 \| \| --- \| | 206 | 66 |  | 0.186 | | 23 |  | | 0.841 | 167 |  | 1.000 | | 11 |  | | 0.505 | |
| GG | 167 | 58 | 109 |  | | 20 | 147 | |  | 135 | 32 |  | | 8 | 159 | |  | |
| GA | 36 | 8 | 28 |  | | 3 | 33 | |  | 29 | 7 |  | | 3 | 33 | |  | |
| AA | 3 | 0 | 3 |  | | 0 | 3 | |  | 3 | 0 |  | | 0 | 3 | |  | |
| rs7278 | 206 | 66 |  | 0.232 | | 23 |  | | 1.000 | 167 |  | 0.337 | | 11 |  | | 1.000 | |
| CC | 165 | 57 | 108 |  | | 19 | 146 | |  | 131 | 34 |  | | 9 | 156 | |  | |
| TC | 37 | 9 | 28 |  | | 4 | 33 | |  | 33 | 4 |  | | 2 | 35 | |  | |
| TT | 4 | 0 | 4 |  | | 0 | 4 | |  | 3 | 1 |  | | 0 | 4 | |  | |
| rs9542 | 206 | 66 |  | 0.200 | | 23 |  | | 0.754 | 167 |  | 0.730 | | 11 |  | | 0.527 | |
| GG | 28 | 5 | 23 |  | | 3 | 25 | |  | 24 | 4 |  | | 0 | 28 | |  | |
| AG | 92 | 33 | 59 |  | | 12 | 80 | |  | 75 | 17 |  | | 6 | 86 | |  | |
| AA | 86 | 28 | 58 |  | | 8 | 78 | |  | 68 | 18 |  | | 5 | 81 | |  | |
| rs8025045 | 206 | 66 |  | 1.000 | | 23 |  | | 0.540 | 167 |  | 0.318 | | 11 |  | | 0.637 | |
| GG | 182 | 59 | 123 |  | | 22 | 160 | |  | 150 | 32 |  | | 11 | 171 | |  | |
| GT | 23 | 7 | 16 |  | | 1 | 22 | |  | 16 | 7 |  | | 0 | 23 | |  | |
| TT | 1 | 0 | 1 |  | | 0 | 1 | |  | 1 | 0 |  | | 0 | 1 | |  | |
| rs1042919 | 206 | 66 |  | 0.148 | | 23 |  | | 0.582 | 167 |  | 0.320 | | 11 |  | | 0.530 | |
| TT | 109 | 40 | 69 |  | | 11 | 98 | |  | 89 | 20 |  | | 8 | 101 | |  | |
| AT | 88 | 22 | 66 |  | | 12 | 76 | |  | 69 | 19 |  | | 3 | 85 | |  | |
| AA | 9 | 4 | 5 |  | | 0 | 9 | |  | 9 | 0 |  | | 0 | 9 | |  | |
| rs851 | 206 | 66 |  | 0.746 | | 23 |  | | 0.723 | 167 |  | 0.359 | | 11 |  | | 0.693 | |
| GG | 14 | 4 | 10 |  | | 2 | 12 | |  | 13 | 1 |  | | 0 | 14 | |  | |
| GA | 73 | 26 | 47 |  | | 9 | 64 | |  | 56 | 17 |  | | 3 | 70 | |  | |
| AA | 119 | 36 | 83 |  | | 12 | 107 | |  | 98 | 21 |  | | 8 | 111 | |  | |
| rs3842 | 206 | 66 |  | 0.518 | 23 | |  | 0.506 | | 167 |  | | 0.182 | 11 | |  | 0.795 |  |
| AA | 116 | 41 | 75 |  | 16 | | 100 |  | | 99 | 17 | |  | 6 | | 110 |  |  |
| AG | 75 | 21 | 54 |  | 6 | | 69 |  | | 57 | 18 | |  | 5 | | 70 |  |  |
| GG | 15 | 4 | 11 |  | 1 | | 14 |  | | 11 | 4 | |  | 0 | | 15 |  |  |
| rs4148380 | 206 | 66 |  | 0.651 | 23 | |  | 0.844 | | 167 |  | | 0.676 | 11 | |  | 0.428 |  |
| GG | 181 | 57 | 124 |  | 21 | | 160 |  | | 148 | 33 | |  | 11 | | 170 |  |  |
| GA | 25 | 9 | 16 |  | 2 | | 23 |  | | 19 | 6 | |  | 0 | | 25 |  |  |
| AA |  |  |  |  | 0 | | 0 |  | |  |  | |  | 0 | |  |  |  |
| rs3743527 | 206 | 66 |  | 0.737 | 23 | |  | 0.454 | | 167 |  | | 0.026 | 11 | |  | 0.534 |  |
| CC | 63 | 22 | 41 |  | 6 | | 57 |  | | 48 | 15 | |  | 2 | | 61 |  |  |
| TC | 108 | 32 | 76 |  | 11 | | 97 |  | | 85 | 23 | |  | 6 | | 102 |  |  |
| TT | 35 | 12 | 23 |  | 6 | | 29 |  | | 34 | 1 | |  | 3 | | 32 |  |  |
| rs212091 | 206 | 66 |  | 1.000 | 23 | |  | 0.869 | | 167 |  | | 0.011 | 11 | |  | 0.185 |  |
| AA | 122 | 39 | 83 |  | 15 | | 107 |  | | 107 | 15 | |  | 5 | | 117 |  |  |
| AG | 72 | 23 | 49 |  | 7 | | 65 |  | | 51 | 21 | |  | 4 | | 68 |  |  |
| GG | 12 | 4 | 8 |  | 1 | | 11 |  | | 9 | 3 | |  | 2 | | 10 |  |  |
| rs212090 | 206 | 66 |  | 0.004 | 23 | |  | 0.716 | | 167 |  | | 0.570 | 11 | |  | 0.138 |  |
| TT | 135 | 37 | 98 |  | 14 | | 121 |  | | 112 | 23 | |  | 9 | | 126 |  |  |
| AT | 63 | 29 | 34 |  | 8 | | 55 |  | | 48 | 15 | |  | 1 | | 62 |  |  |
| AA | 8 | 0 | 8 |  | 1 | | 7 |  | | 7 | 1 | |  | 1 | | 7 |  |  |
| rs10517 | 206 | 66 |  | 0.700 | 23 | |  | 0.628 | | 167 |  | | 0.750 | 11 | |  | 0.580 |  |
| CC | 92 | 28 | 46 |  | 12 | | 80 |  | | 74 | 18 | |  | 4 | | 88 |  |  |
| CT | 95 | 33 | 62 |  | 10 | | 85 |  | | 76 | 19 | |  | 7 | | 88 |  |  |
| TT | 19 | 5 | 14 |  | 1 | | 18 |  | | 17 | 2 | |  | 0 | | 19 |  |  |
| rs9024 | 206 | 66 |  | 0.861 | 23 | |  | 1.000 | | 167 |  | | 0.086 | 11 | |  | 0.007 |  |
| GG | 123 | 38 | 85 |  | 14 | | 109 |  | | 101 | 22 | |  | 2 | | 121 |  |  |
| GA | 69 | 23 | 46 |  | 8 | | 61 |  | | 52 | 17 | |  | 7 | | 62 |  |  |
| AA | 14 | 5 | 9 |  | 1 | | 13 |  | | 14 | 0 | |  | 2 | | 12 |  |  |
