# Supplementary figures and images for: Polymorphisms at microRNA binding sites of Ara-C and anthracyclines-metabolic pathway genes are associated with outcome of acute myeloid leukemia patients
Source: J Transl Med. 2017 Nov 15;15:235. doi: 10.1186/s12967-017-1339-9 (PMC5688732; doi:10.1186/s12967-017-1339-9)

Figure S1


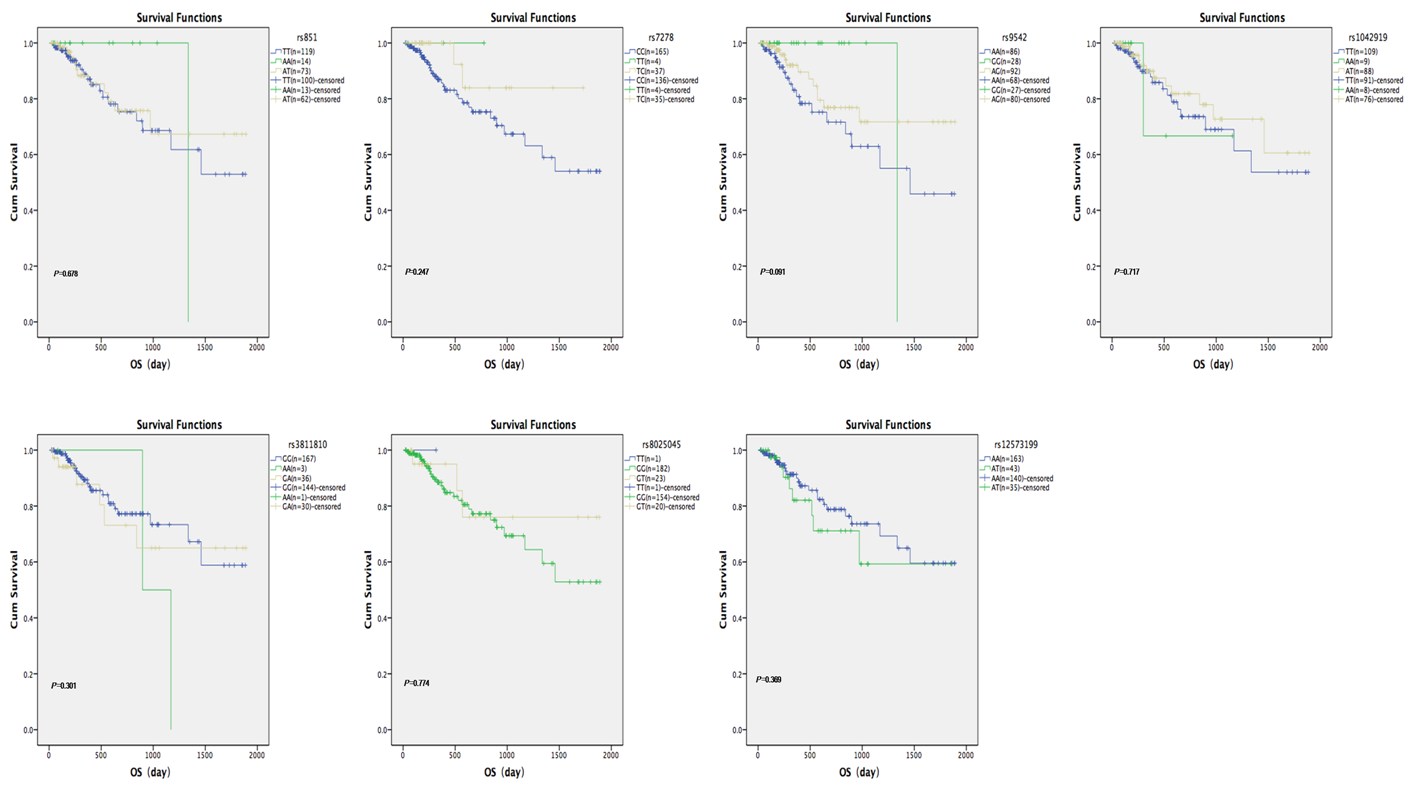


Figure S2


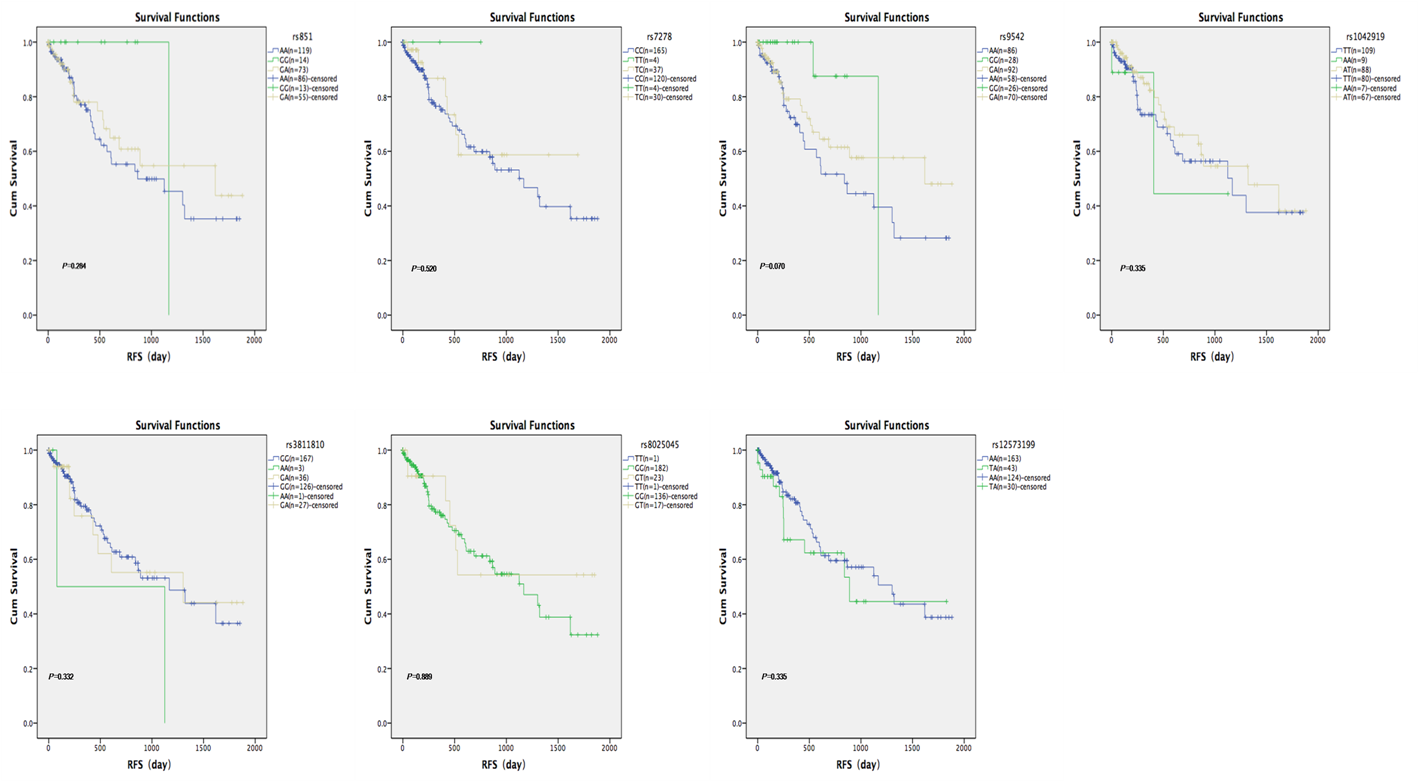


Figure S3


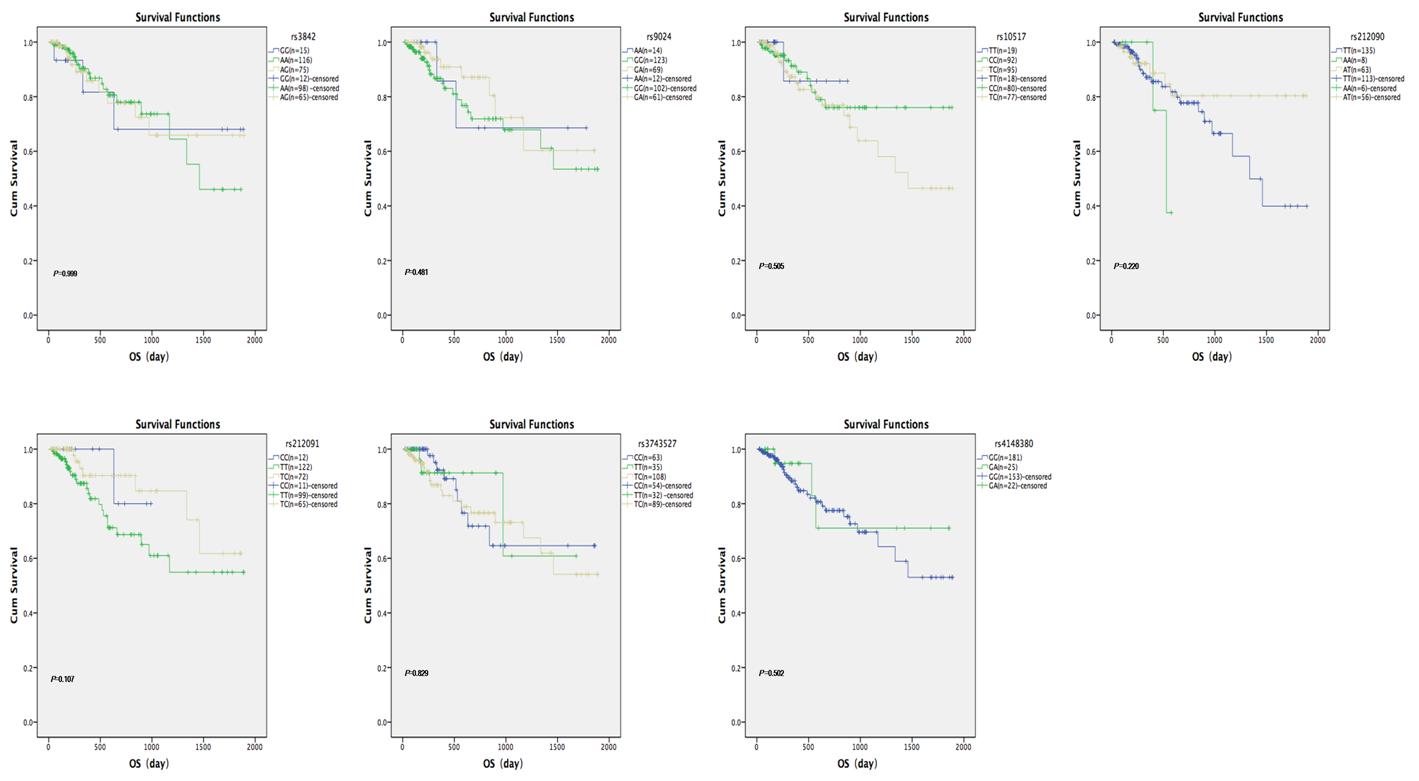


Figure S4

Supplement: Supplementary file 7 — Additional file 7: Figure S1. Kaplan–Meier evaluation of associations between NT5C2 rs12573199, DCTD rs7278, rs3811810, rs851 and rs9542, SLC28A1 rs8025045, and RRM1 rs1042919 polymorphisms with OS in AML patients. Figure S2. Kaplan–Meier evaluation of associations between NT5C2 rs12573199, DCTD rs7278, rs3811810, rs851and rs9542, SLC28A1 rs8025045, and RRM1 rs1042919 polymorphisms with RFS in AML patients. Figure S3. Kaplan–Meier evaluation of associations between rs9024, rs3842, rs212090, rs212091, rs3743527, rs4148380, rs10517 polymorphisms with OS in AML patients. Figure S4. Kaplan–Meier evaluation of associations between rs9024, rs3842, rs212090, rs212091, rs3743527, rs4148380, and rs10517 polymorphisms with RFS in AML patients. [file 12967_2017_1339_MOESM7_ESM.docx]
