# Supplementary material for: Polymorphisms at microRNA binding sites of Ara-C and anthracyclines-metabolic pathway genes are associated with outcome of acute myeloid leukemia patients
Source: J Transl Med. 2017 Nov 15;15:235. doi: 10.1186/s12967-017-1339-9 (PMC5688732; doi:10.1186/s12967-017-1339-9)
Supplement: Supplementary file 8 — Additional file 8: Table S7. Combined effects of rs10786736, rs3734703 and rs8139 genotypes on survival of AML patients. [file 12967_2017_1339_MOESM8_ESM.docx]

**Table S7. Combined effects of rs10786736, rs3734703 and rs8139** **genotypes on survival of AML patients**

| **Endpoint** | **Composite Scorea Scorea Scorea** | **N** | ***P*b** | **HR (95% CI) b** |
| --- | --- | --- | --- | --- |
| **OS** | 0 | 16 |  | 1.00 (reference) |
|  | 1 | 47 | 0.108 | 0.438（0.160-1.200） |
|  | 2 | 78 | 0.013 | 0.294（0.112-0.769） |
|  | 3 | 65 | 0.000 | 0.085（0.025-0.296） |
|  | 0+1 | 63 |  | 1.00 (reference) |
|  | 2+3 | 143 | 0.001 | 0.316（0.155-0.642） |
| RFS | 0 |  |  | 1.00 (reference) |
|  | 1 | 16 | 0.444 | 0.713（0.300-1.695） |
|  | 2 | 47 | 0.027 | 0.385（0.165-0.899） |
|  | 3 | 78 | 0.002 | 0.237（0.097-0.583） |
|  | 0+1 | 63 |  | 1.00 (reference) |
|  | 2+3 | 143 | 0.001 | 0.389（0.224-0.675） |

**a**Combined genotype score model was created by compiling the genotyped data of SNPs rs10786736, rs3734703 and rs8139. Score 1 indicated favorable genotypes (i.e.， rs10786736 CC/CG genotype, rs3734703 CC genotype, or rs8139 TT/CT genotype) and a score of 0 indicated unfavorable genotypes (i.e. rs10786736 GG genotype, rs3734703 AA/CA genotype, or rs8139 CC genotype). After adding up these scores, four composite score groups were generated: composite score 0, 1, 2 and 3.

**b**Adjusted for risk stratifications.
